# Psychometric comparison of CHU9D and PedsQL 4.0 proxy version administered to parents of children with congenital colorectal conditions in Australia

**DOI:** 10.1007/s10198-025-01797-0

**Published:** 2025-06-05

**Authors:** Tianxin Pan, Misel Trajanovska, Nathan Kwong, Sebastian K. King, Ilias Goranitis

**Affiliations:** 1 Economics of Genomics and Precision Medicine Unit, Centre for Health Policy, Melbourne School of Population and Global Health, The University of Melbourne, 207 Bouverie Street, Melbourne, VIC 3010 Australia; 2 Murdoch Children’s Research Institute, Melbourne, VIC Australia; 3 Department of Paediatric Surgery, The Royal Children’s Hospital, Melbourne, VIC Australia; 4 Department of Paediatrics, The University of Melbourne, Melbourne, VIC Australia; 5 Australian Genomics, Murdoch Children’s Research Institute, Melbourne, VIC Australia

**Keywords:** Health-related quality of life, Psychometric comparison, CHU9D, PedsQL, Colorectal conditions, Paediatrics

## Abstract

**Objective:**

To assess the psychometric performance of the Child Health Utility (CHU9D) proxy version compared with the Pediatric Quality of Life Inventory (PedsQL) in Australian children aged 0–7 years with anorectal malformations (ARM) or Hirschsprung disease (HD).

**Methods:**

Parents of children with ARM or HD were identified from a patient database managed by a tertiary paediatric hospital in Australia over the past 20 years. Since 2020, CHU9D and PedsQL proxy report versions were administered to parents via telephone interview. Using data collected between 2020 and 2022, we assessed the feasibility, ceiling and floor effects, known-group validity and convergent validity for both instruments in the total sample, by conditions and child age.

**Results:**

The study included 145 children with ARM or HD, among which, 13.1% had missing values on the CHU9D schoolwork dimension, and 20.7% had missing values on the PedsQL school functioning domain (2–4 year old version). The CHU9D and PedsQL did not demonstrate ceiling effects. The CHU9D showed stronger effect size (ES) in differentiating children with ARM (ES = 0.32) or HD (ES = 0.90) with healthy children compared to the PedsQL. We did not find statistically significant differences in CHU9D or PedsQL scores between ARM and HD. There were moderate to strong correlations in most theoretically related dimensions of the CHU9D and PedsQL.

**Conclusion:**

The CHU9D and PedsQL demonstrated comparable and acceptable psychometric properties in Australian children aged 2 years and above with ARM or HD. However, the validity of the CHU9D in children under 2 years old needs to be further explored and modification may be needed.

**Supplementary Information:**

The online version contains supplementary material available at 10.1007/s10198-025-01797-0.

## Introduction

Complex congenital colorectal conditions, such as anorectal malformations (ARM) and Hirschsprung disease (HD) are rare disorders occurring in 1 in 5,000 births [1, 2]. They are characterised by abnormal development of the anus and rectum [3, 4], which often requires surgical intervention early in life. Despite advances in surgical care, patients continue to face ongoing challenges across different stages of life. In early childhood, they may experience delays in toilet training and need ongoing bowel management to help symptoms of constipation, diarrhoea, and incontinence [5]. During adolescence, they may experience problems with anxiety, peer rejection, behavioural problems, low self-esteem, body image and sexual health concerns [6, 7]. In young adulthood, most patients will need to navigate transition to adult healthcare, where several challenges exist in the continuity and coordination of care. Studies have also reported patients experience academic difficulties, limitations in sexual function and reproductive concerns [6, 7].

In addition to functional problems such as persistent constipation and faecal incontinence, bladder control problems, and recurrent enterocolitis [5], an increasing number of studies have examined the health-related quality of life (HRQOL) of children affected by ARM or HD [8, 9]. For example, some studies have reported increased psychological distress and frequent school absence for children with ARM or HD compared to their peers [5, 10], while others found no evidence of lower HRQOL compared to their healthy counterparts [11–13].

Most studies have focused on the exploration of HRQOL during adolescence and into adulthood [14–19], with limited evidence in infants and children under 5 years of age [20]. Anecdotally, children with ARM or HD are most vulnerable during the early years from diagnosis to school start, where they require a high level of healthcare and management support. Adverse events in childhood such as illness have been found to have long-term and enduring consequences on health and well-being in later life and overall life trajectory [21, 22]. Investigation of HRQOL for ARM or HD children in their early years can contribute to our understanding of the impact across the life course and provide a reference base for relevant health interventions targeted at this population. Exploration of whether there are differences between these two colorectal conditions is also important, where existing studies either combined ARM and HD patients as one cohort or focused on one condition. Last but not least, most existing studies examined HRQOL of colorectal conditions via disease-specific measures, such as the Gastrointestinal Quality of Life Index [14], and Hirschsprung and Anorectal Malformation Quality of Life (HAQL) questionnaire [23–25]. These measures are specially designed for colorectal conditions and can be used to measure the severity of the disease in clinical settings. However, the outcomes are not comparable across different conditions. On the other hand, generic HRQOL measures, which are standardised multi-dimensional instruments designed to assess overall health status [26], are sensitive in different conditions [26], enabling comparison across different populations and interventions, particularly for health economic evaluations [27]. One of the generic measures, the Pediatric Quality of Life Inventory (PedsQL) [5, 8], has been widely used in clinical settings and monitoring general population health in children. The PedsQL has been validated for use in children with HD from birth to 15 years [5, 20] and children with ARM aged 17 months to 18 years [8, 28], which could be used as a comparison in validation studies for generic HRQOL instruments in this cohort. Nonetheless, the PedsQL is not currently accompanied by preference weights, which limits its use in estimating quality-adjusted life years (QALYs) for economic evaluation and resource allocation decisions.

More recently, several new paediatric HRQOL instruments have been developed for measuring child health that are accompanied by preference weights, for example the Child Health Utility 9D (CHU9D), EQ-5D-Y-3L, and Health Utilities Index (HUI2) [29, 30]. The CHU9D is a concise generic HRQOL instrument originally developed for older children aged 7 to 17 years, which has been validated in various clinical conditions [31, 32]. More recent studies have explored using a proxy version administered to parents and caregivers for children aged 6 to 7 years [33] and under 5 years [34]. One study in Australia found it to be valid and reliable to measure HRQOL in children aged 2 to 4 years who were healthy and those with a health condition [35]. Nevertheless, no study has evaluated the psychometric performance of the CHU9D in children aged 5 to 7 years and under 5 years with ARM or HD.

This study aimed to assess the psychometric performance of the CHU9D proxy version compared to the PedsQL in children aged 0 to 7 years with ARM or HD.

## Methods

### Data source

This study used data collected from an ongoing large annual longitudinal study (the ColoQol Study), which commenced in 2019. Eligible participants were identified from an existing Colorectal Database managed by a tertiary paediatric hospital in Melbourne Australia over the past 20 years. Families of patients ( < = 7 years) with ARM or HD who had undergone major surgical repair (i.e., reconstruction surgery for ARM and pull-through surgery for HD patients) were contacted and invited to participate in the ColoQol Study. One parent of each family who consented to participate in the study completed the questionnaire over telephone with trained interviewers. Detailed information on recruitment and administration has been described elsewhere [1, 36]. Ethics approval was granted by the Human Research Ethics Committee at The Royal Children’s Hospital Melbourne (Ethics approval number: HREC 36003).

### Sample

The CHU9D questionnaire was administered to parents since 2020. We used three waves of data available to us from 2020 to 2022. This study used each participant’s baseline data among those who completed both the CHU9D and the age-matched PedsQL versions.

### Survey questionnaire and HRQOL instrument

The ColoQol survey consisted of the following parts (fixed order): (1) child demographics; (2) parent demographics; (3) parent reported outcomes including the Family Management Measure (FaMM) [37], Parent Experience of Child Illness (PECI) [38], SF-12 [39], the Patient-Reported Outcomes Measurement Information System (PROMIS) Anxiety and PROMIS Depression short-forms [40]; (4) age-specific PedsQL 4.0 core generic version [41]; (5) health service use and productivity cost questionnaire; (6) Parent wellbeing impacts and AQol-8D [42]; (7) child health utility instruments: EQ-5D-Y-3L [43]and CHU9D. The questionnaires were administrated to parents over telephone with trained interviewers. This study reports data from the CHU9D and PedsQL.

### CHU9D

**Fig. 1:**
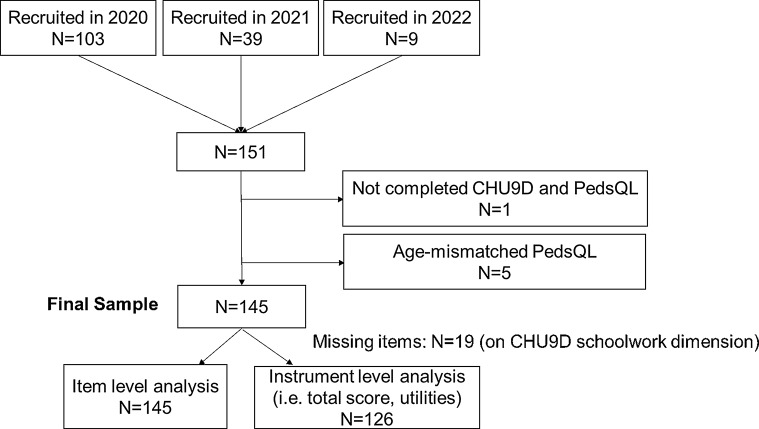
Study sample inclusion flow

The CHU9D proxy report version (English) was used in the study. The CHU9D uses the recall period of ‘today’. It consists of nine dimensions: worried, sad, pain, tired, annoyed, schoolwork/homework, sleep, daily routine, and able to join in activities [44]. Each dimension has five levels ranging from level 1 (no problems/not at all) to level 5 (couldn’t do this at all, very). Considering the relevance of schoolwork and homework in infants and toddlers, interviewers asked parents to think about their child’s abilities with learning tasks and activities as appropriate for their age. To calculate health utility scores, we used the value set based on the preferences of Australian adolescents [45]. We also used the value set based on the preferences of UK general adults in sensitivity analysis [46]. Participants who had missing data for an individual dimension of the CHU-9D were included in the dimension level analysis, but excluded from the calculation of utility values (details provided in Fig. 1).

### PedsQL

The PedsQL 4.0 core generic version (parent report, English version) for infants aged 0–12 months, 13–24 months, toddlers aged 2–4 years and children aged 5–7 years was used in this study [41]. The PedsQL uses the recall period of ‘past one month’. The PedsQL for infants (1–12 months and 13–24 months) includes 36 and 45 items respectively, covering five domains: physical functioning (PF), physical symptoms (PS), emotional functioning (EF), social-functioning (SF) and cognitive-functioning (CF). The PedsQL for toddlers (2–4 years) and for children (5–7 years) includes 21 and 23 items respectively, covering PF, EF, SF and school functioning (SchF). Each item has a raw score ranging from 0 (never a problem) to 4 (always a problem), which is converted to a 0 to 100 scale as follows: 0 = 100, 1 = 75, 2 = 50, 3 = 25, 4 = 0 [47]. Domain level summary scores were calculated by the summation of item scores (on a 0-100 scale) over the number of items in each domain. The total PedsQL score was calculated as the sum of the domain scores divided by the number of domains. If one domain has more than half of the items missing, the domain score was not computed. The total PedsQL score was calculated as the sum of all the items over the number of items answered on all the domains [47]. A higher PedsQL score suggests better HRQOL.

### Data analysis

Descriptive statistics were used to summarize the characteristics of patients and their parents (i.e. participants included in the study). We assessed feasibility by calculating the proportion of missing values for each item of each instrument in the total sample, by conditions (ARM or HD) and by age (1–24 months, 2–4 years and 5–7 years).

At the item/dimension level, we examined response distribution by reporting the frequency of responses to each level in CHU9D dimensions and PedsQL items and presented the distribution in figure. We compared the differences by condition in both instruments and by age groups in CHU9D, using fisher’s exact test. At the instrument level, we reported means and standard deviations (SD) of PedsQL domain scores and total scores and CHU9D utility scores for the total sample and by condition and by age.

The ceiling and floor effects were assessed at the instrument level, by reporting the proportion of choosing the highest and lowest levels across all dimensions for CHU9D, and by reporting the proportion of a score of 100 or a score of 0 on PedsQL total scores. The analysis was performed in the total sample and by conditions and ages. Following literature, the 15% threshold was used for examining ceiling and floor effect [48, 49].

Known-group validity assesses the extent to which an instrument detects differences between groups where responses might be expected to differ. Group differences were assessed at the instrument level by comparing CHU9D utilities and PedsQL total scores, and at dimension level by comparing CHU9D dimension responses and PedsQL domain scores. Given the nature of condition progression, we assessed the known-group validity by age. Though it is unclear whether HRQOL differs between ARM and HD from the literature, we hypothesized that there would be differences and examined the differences between the two conditions. We further compared differences by severity within each condition. We categorized ARM patients based on their ARM types (simple or complex). Anal stenosis, anorectal malformation with no fistula, anterior anus, perineal fistula, rectal atresia, rectourethral fistula-bulbar, urogenital sinus, vestibular fistula were considered ‘simple’. Rectourethral fistula-prostatic, rectovaginal fistula, rectovesical or bladderneck fistula and cloaca were considered ‘complex’ [50, 51]. We categorized HD patients by length of segment (short segment versus long segment or total colonic). In addition, we compared ARM and HD in children age 2 years and above with corresponding scores from healthy controls in Australia respectively [13]. The statistical significance of the difference across groups in our sample was tested using nonparametric Mann–Whitney U test or Kruskal-Wallis test as the summary scores were not normally distributed. The magnitudes of the difference across groups were assessed using Cohen’s D effect size. Effect sizes of 0.2–0.49 were considered small, 0.5–0.79 moderate, and ≥ 0.8 large [52, 53].

Convergent validity assesses whether a dimension of the instrument correlates appreciably with dimensions that should be related to it in theory [54]. We assessed the correlations at the following levels: between CHU9D dimensions and PedsQL items, CHU9D dimensions and PedsQL domain scores, and between CHU9D utilities and PedsQL total scores. Based on literature and priori consensus via expert opinions [55], we hypothesized moderate to strong correlations between items measuring similar constructs (e.g. tired versus low energy level and feeling tired, worried versus feeling afraid or scared). The analysis was performed in the total sample. Correlations were calculated using Spearman’s correlation. Correlations of 0.1–0.29 were considered weak, 0.3–0.49 moderate, and ≥ 0.5 strong [53].

## Results

### Sample characteristics

There were 103, 39 and 9 participants who completed their baseline survey in 2020, 2021 and 2022 respectively. Among the 151 participants, one did not complete the PedsQL, and another five parents were given age mismatched PedsQL versions thus they were excluded. The final sample included 145 patients aged 0 to 7 years with ARM or HD. Figure 1 shows the study sample inclusion flow.

As shown in Table 1, of 145 patients with congenital colorectal conditions, 88 (60.7%) were diagnosed with ARM, and nearly half of the total sample were diagnosed within the first day of birth. The majority of children were male (69.7%), with varied proportions representing each age category (1–24 months 35.9%, 2–4 years 40.0%, and 5–7 years 24.1%) at the time of interview. Among parents, 73.8% had a bachelor’s degree or above, 55.9% were employed full-time or part-time, while 24.1% were a full-time carer.

**Table 1:** Sample characteristics

	ARM		HD		Total	
Year	N	%	N	%	N	%
2020	67	76.1%	36	63.2%	103	71.0%
2021	17	19.3%	18	31.6%	35	24.1%
2022	4	4.6%	3	5.3%	7	4.8%
Child’s age at interview						
0	19	21.6%	11	19.3%	30	20.7%
1	16	18.2%	6	10.5%	22	15.2%
2	17	19.3%	10	17.5%	27	18.6%
3	6	6.8%	7	12.3%	13	9.0%
4	14	15.9%	4	7.0%	18	12.4%
5	13	14.8%	11	19.3%	24	16.6%
6	3	3.4%	6	10.5%	9	6.2%
7	0	0.0%	2	3.5%	2	1.4%
Child’s gender						
Female	37	42.1%	7	12.3%	44	30.3%
Male	51	58.0%	50	87.7%	101	69.7%
Age at diagnosis						
at birth	40	46.5%	1	1.8%	41	28.9%
first day	24	27.9%	3	5.4%	27	19.0%
first week	13	15.1%	38	67.9%	51	35.9%
first month	3	3.5%	5	8.9%	8	5.6%
first 6 months	3	3.5%	5	8.9%	8	5.6%
other	3	3.5%	4	7.1%	7	4.9%
Child’s Birth country						
Australia	86	97.7%	55	96.5%	141	97.2%
Other	2	2.3%	2	3.5%	4	2.8%
Caregiver’s gender						
Female	78	88.6%	51	89.5%	129	89.0%
Male	10	11.4%	6	10.5%	16	11.0%
Caregiver’s birth country						0.0%
Australia	62	70.5%	44	77.2%	106	73.1%
Other	26	29.6%	13	22.8%	39	26.9%
Caregiver’s highest level of education						
Postgraduate Qualification or above	17	19.3%	11	19.3%	28	19.3%
Completed a University	52	59.1%	27	47.4%	79	54.5%
Some University/TAFE	6	6.8%	5	8.8%	11	7.6%
Finished high school	9	10.2%	9	15.8%	18	12.4%
Did not finish high school	4	4.6%	5	8.8%	9	6.2%
Caregiver’s employment status						
Employed full-time	20	22.7%	10	17.5%	30	20.7%
Employed part-time	30	34.1%	21	36.8%	51	35.2%
Home duties/Full-time carer	20	22.7%	15	26.3%	35	24.1%
Paternity leave	13	14.8%	10	17.5%	23	15.9%
Unemployed	5	5.7%	1	1.8%	6	4.1%
Caregiver’s relationship status						
Married/partnered	83	94.3%	51	89.5%	134	92.4%
Re-partnered	1	1.1%	0	0.0%	1	0.7%
Single	2	2.3%	6	10.5%	8	5.5%
Separated/divorced	2	2.3%	0	0.0%	2	1.4%

ARM: anorectal malformations; HD: Hirschsprung disease

### Feasibility

For the CHU-9D, 19 parents of children among 145 participating parents (13.1%) did not provide a response to the schoolwork dimension, with 23.1% missing in children aged 1–24 months and 12.1% missing in children aged 2–4 years. For PedsQL, there was no missing data among children aged 1–24 months, but 20.7% and 3.9% of parents of children aged 2–4 years and 5–7 years had more than half of the items missing on the school functioning domain respectively. Detailed information is provided in electronic supplementary material (ESM) Table S1.

### Response distribution

**Fig. 2:**
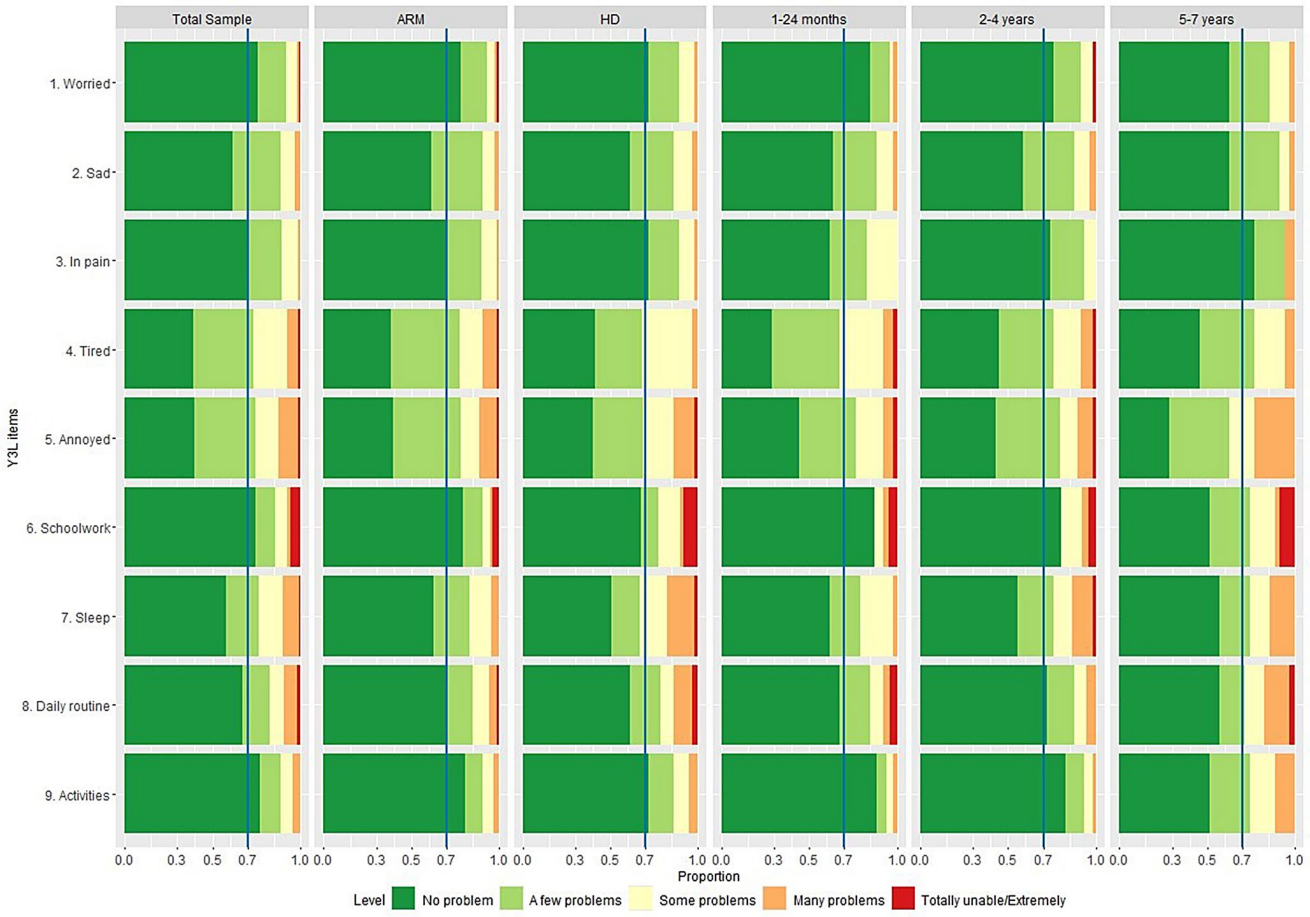
Response distribution on CHU9D dimensions in the total sample and by subgroups. Legend: ARM: anorectal malformations; HD: Hirschsprung disease

Figure 2 shows the response distribution for CHU9D. In our sample, 70.7% and 60.0% of parents reported at least a few problems (level 2) for ‘tired’ and ‘annoyed’, more than the other seven dimensions (ranging from 22.7% to 42.1%). There were statistically significant differences in the response distribution for ‘pain’, ‘schoolwork’ and ‘activities’ dimensions across the three age groups, but the distribution pattern was similar in ARM and HD groups (ESM Tables S2 and S3).

Figures S1-4 in the supplementary materials show the distribution of PedsQL items for each age group. We did not find statistically significant differences in the PedsQL response distribution between HD and ARM, except for two PedsQL items on EF for children aged 1–24 months (‘feeling angry’ in 1–12 months; ‘crying a lot’ and ‘crying or fussing when left alone’ in 13–24 months), and three PedsQL items for children aged 2–4 years (‘feeling sad’, ‘having aches or pains’ and ‘helping to pick up toys’).

As shown in Table 2, ceiling effects were not present for CHU9D in the total sample (8.7%), ARM sample (6.5%) or HD sample (12.2%). The PedsQL did not demonstrate ceiling effects in any sample, with only 0.8%, 0%, and 2% of respondents reporting best levels for all items respectively. No floor effects were found for any sample. The mean and standard deviation (SD) of CHU9D utilities and PedsQL scores were 0.73 [0.21] and 77.77 [15.56] respectively.

**Table 2:** Ceiling effects, floor effects and total scores

	Total sample	ARM	HD	1–24 months	2–4 year	5–7 years
	*N* = 126	*N* = 77	*N* = 49	*N* = 40	*N* = 51	*N* = 35
CHU9D ceiling	8.7%	6.5%	12.2%	12.5%	5.9%	8.6%
CHU9D floor	0%	0%	0%	0%	0%	0%
CHU9D utilities	0.73(0.21)	0.75(0.19)	0.7(0.23)	0.76(0.2)	0.74(0.19)	0.68(0.23)
PedsQL ceiling	0.8%	0%	2%	0%	2%	0%
PedsQL floor	0%	0%	0%	0%	0%	0%
PF	79.61(19.65)	79.78(19.52)	79.33(20.05)	76.08(17.93)	83.09(18.33)	78.57(22.87)
PS	77.25(12.81)	77.05(13.23)	77.71(12.31)	77.25(12.81)		
EF	70.04(17.42)	71.98(15.21)	66.99(20.2)	67.5(15.39)	73.82(17.02)	67.43(19.53)
SF	85.16(18.65)	87.66(15.96)	81.22(21.82)	90.25(12.57)	87.16(18.12)	76.43(22.35)
CF	82.71(20.55)	83.43(22.68)	81.02(15.17)	82.71(20.55)		
School	74.46(22.36)	77.62(21.2)	70.56(23.43)		77.18(22.17)	71.1(22.46)
Total	77.77(15.56)	79.4(14.18)	75.19(17.35)	76.41(12.29)	81.3(14.87)	74.17(18.91)

ARM: anorectal malformations; HD: Hirschsprung disease. PF: physical functioning; PS: physical symptoms; EF: emotional functioning; SF: social-functioning; CF: cognitive-functioning; School: School functioning

### Known-group validity

Table 3 summarises the known-group validity with CHU9D utilities and PedsQL scores. The CHU9D utilities and PedsQL scores were lower in HD sample (0.70 [0.23] and 75.19 [17.35]) compared to those in ARM (0.75 [0.19] and 79.4 [14.18]). We did not find statistically significant differences in mean scores by conditions (i.e., ARM versus HD), and by severity level within condition. In terms of the magnitude of differences, we found small effect sizes for both instruments differentiating children with ARM or HD (ES = 0.23 for CHU9D utilities and 0.27 for PedsQL), and PedsQL differentiating severity level in HD patients based on length of segment (ES = 0.37). We found small effect sizes on CHU9D and PedsQL in differentiating children with different ages. CHU9D was more sensitive in differentiating children aged 5–7 years with 1–24 months (ES = 0.38) while PedsQL was more sensitive in differentiating children aged 2–4 years with 1–24 months (ES = 0.36). The results were similar when using CHU9D UK value set (ESM Table S4).

**Table 3:** Known group validity of CHU9D utilities and PedsQL total scores for different groups

Groups	Sample size	CHU9D utilities					PedsQL total scores				
		Mean	SD	Difference	*p*-value	Cohen’s d ES	Mean	SD	Difference	*p*-value	Cohen’s d ES
Clinical conditions											
ARM	77	0.75	0.19	-0.05	0.389	0.23	79.40	14.18	-4.21	0.206	0.27
HD	49	0.7	0.23				75.19	17.35			
ARM type											
Simple	56	0.75	0.2	-0.01	0.576	0.03	79.09	13.97	1.14	0.572	0.08
Complex	21	0.75	0.18				80.23	15.05			
HD length of segment											
Short	15	0.68	0.22	0.04	0.461	0.15	70.60	18.85	6.61	0.242	0.37
long segment or total colonic	34	0.71	0.24				77.22	16.54			
Age group											
1–24 month(ref)	40	0.76	0.2		0.054		76.41	12.29		0.093	
2–4 years	51	0.74	0.19	-0.02		0.08	81.30	14.87	4.89		0.36
5–7 years	35	0.68	0.23	-0.08		0.38	74.17	18.91	-2.24		0.14

Standard thresholds 0.2 to < 0.5, 0.5 to < 0.8, and 0.8 or more denote small, medium, and large effect sizes, respectively. Known group validity: *n* ≥ 100 per group very good; *n* = 50–99 per group adequate; *n* = 30–49 per group doubtful; *n* < 30 per group inadequate

ARM: anorectal malformations; HD: Hirschsprung disease. SD: standard deviation

**Table 4:**
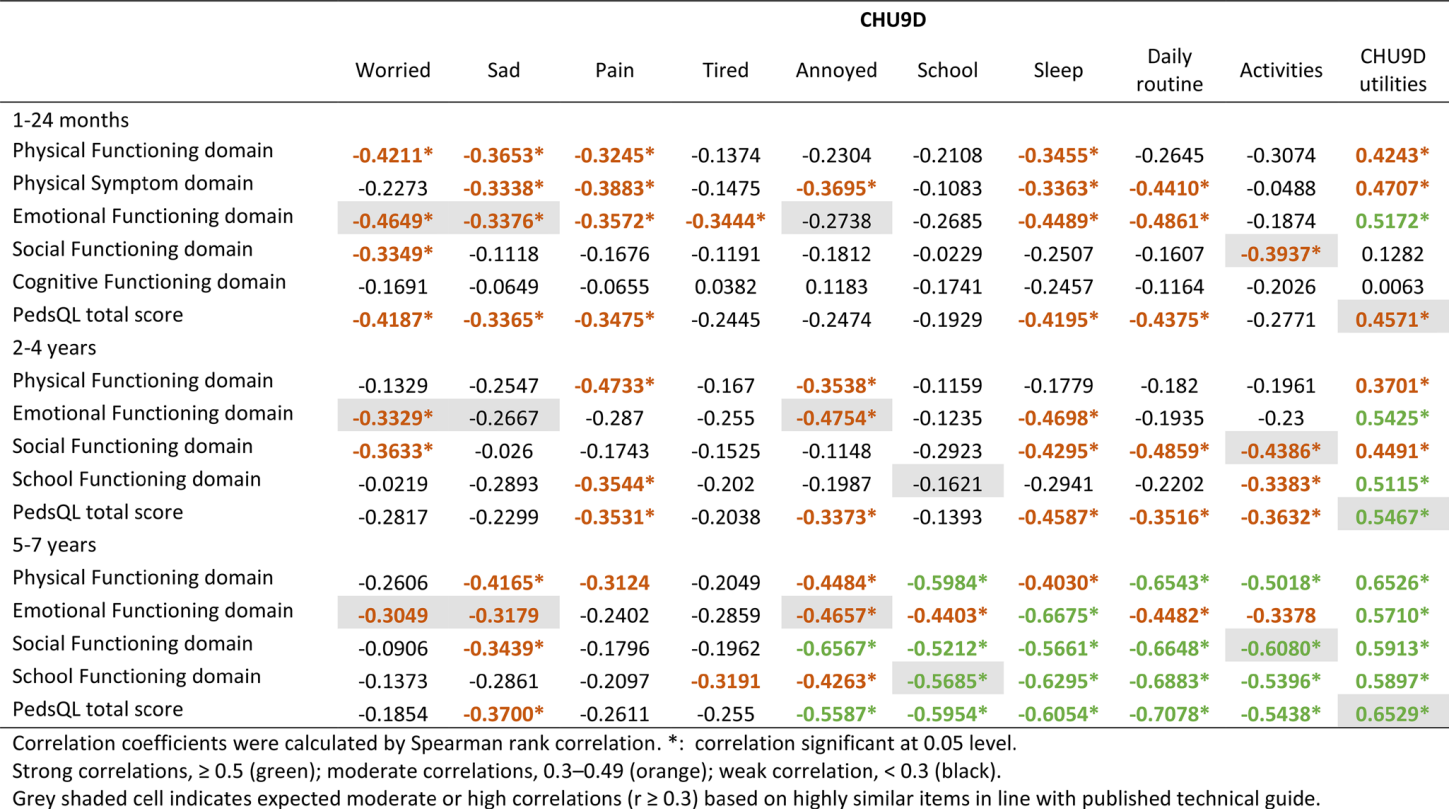
Convergence between CHU9D and PedsQL at dimension and instrument level in the total sample

The effect sizes varied across CHU9D and PedsQL dimensions (ESM Tables S5 and S6). For example, we observed a statistically significant and small effect size in the ‘sleep’ dimension of CHU9D, and non-significant small effects in ‘schoolwork’, ‘daily routine’ and ‘activities’ of CHU9D dimensions and EF, SF and School domains of PedsQL between HD and ARM. Both instruments were more sensitive in differentiating within HD groups compared to ARM groups, as evident by larger effect sizes.

As shown in ESM Table S7, among the children aged 2–4 years, CHU9D showed moderate effect size in differentiating children affected by ARM with healthy controls (ES = 0.32) whereas no relevant effect size was observed on PedsQL scores (ES = 0.10). CHU9D and PedsQL showed moderate and large effect size in differentiating between HD with healthy controls (ES = 0.90 and 0.36 respectively).

### Convergent validity

Table 4 shows the convergence between CHU9D and PedsQL at the dimension and instrument level in the total sample. As hypothesized, we found strong correlations between CHU9D utilities and PedsQL total scores among children aged 2 years and above, and moderate correlations among children under 2 years. At the dimension level, CHU9D and PedsQL showed moderate correlations across all hypothesized correlated domain pairs, except for ‘annoyed’ and EF in children under 2 years, ‘sad’ and EF, ‘schoolwork’ and SchF in children aged 2–4 years. In addition, we also found strong correlations between CHU9D ‘sleep’, ‘daily routine and PedsQL SF and SchF among children 5–7 years, where correlations were not hypothesized.

At the item level, among the CHU9D and PedsQL item pairs hypothesised to be correlated, 5 of 8 (62.5%), 3 of 7 (42.9%), 11 of 17 (64.7%) and 15 of 19 (78.9%) pairs were moderate-to-strongly correlated in age 1–12 months, 13–24 months, 2–4 years and 5–7 years respectively. Sleep and having trouble sleeping showed strong correlations in all age groups (ESM Tables S8, S9, S10 and S11).

## Discussion

This study presents new evidence on the feasibility and validity of CHU9D compared to PedsQL in children with colorectal conditions under 7 years old in Australia. Our study demonstrates feasibility for the CHU9D proxy report and showed comparable psychometric performance when compared with PedsQL, a more widely used generic non-preference accompanied instrument in this condition group.

We found 12.1% and 20.7% of parents of children above 2 years had missing responses to CHU9D schoolwork dimension and PedsQL’s school functioning domain, respectively. The results are comparable with existing studies [33, 56–58]. For children under 2 years old, 21.3% of parents had missing response to CHU9D schoolwork dimension while PedsQL did not include school related items for children in this age group. There were limited validation studies for the CHU9D in children under 5 years old; most studies did not permit missing responses on CHU9D [35, 59] while acknowledging it limited the assessment of feasibility as it may lead to respondents to randomly select an answer. Nevertheless, our results showed that CHU9D had similar feasibility compared to PedsQL in children aged 2 years and above with colorectal conditions. Further research on feasibility of CHU9D in children under 2 years and adaption of the schoolwork domain may be needed.

CHU9D did not exhibit ceiling effects in our sample and by condition and age groups, which is expected given the severity of the conditions and impacts on children’s HRQOL. At the dimension/item level, for both CHU9D and PedsQL, more problems were reported on mental and emotional domains than physical domains; and the pattern was similar in ARM and HD and across infants, toddlers and children, suggesting that children with colorectal conditions were more impacted on mental and emotional aspects compared to physical aspects [10, 20, 60]. In addition, we observed high prevalence of problems in specific PedsQL items in physical domains, for example ‘spitting up after eating’ among children aged 1–12 months (72.7%), ‘having hurts or aches’ among children aged 13–24 months (84.2%), and ‘having gas’ among children aged 1–24 months (73.7%-78.8%), reflecting the nature of the condition.

CHU9D utilities and PedsQL total scores demonstrated similar performance in differentiating between colorectal conditions, with healthy children, by types within each condition and by age groups. Both instruments were able to discriminate between clinical groups with healthy children, with stronger effect sizes on CHU9D utilities. Both instruments were more sensitive in discriminating HD with healthy children compared to discriminating ARM and healthy children. However, between ARM and HD, CHU9D and PedsQL showed small effect sizes but the differences in scores were non-significant. Only one study examined differences between the two conditions using the condition specific instrument HAQL, where they did not find the HAQL’s overall score could discriminate between ARM and HD [61]. This may suggest no differences in HRQOL should be expected between ARM and HD and whether it can be used as ‘known’ group should be further explored. Within each condition, we did not find statistically significant differences by type of ARM or length of segment in HD, which is consistent with literature [11–13]. We only found a small effect size for PedsQL in differentiating HD based on length of segment. However, it is noteworthy that our sample size for different types within ARM or HD was small and considered inadequate.

We found small effect sizes on CHU9D and PedsQL in differentiating children with different ages and the differences were statistically significant at 10%. It is expected given the condition progression and challenges children face at different ages [6, 7, 62]. CHU9D was more sensitive in differentiating children aged 5–7 years with 1–24 months while PedsQL more sensitive in differentiating children aged 2–4 years with 1–24 months. This may be explained by the age of which the instruments were designed for. CHU9D was originally developed for older children 7–17 years. The descriptive system and dimensions were more relevant for older children (e.g. schoolwork, activities) but less relevant for infants. PedsQL has age-specific versions, where items included are different for children under and above 2 years old, but very similar for 2–4 years compared to 5–7 years.

We found moderate to strong correlations in most theoretically related dimensions of CHU9D and PedsQL, particularly in the age group 5–7 years indicating their respective convergent validity. Nevertheless, we only found weak correlations between some emotional dimensions of CHU9D and PedsQL EF domain, evidence of which is mixed in the literature [35, 58, 63]. We found weak correlations between ‘annoyed’ for the CHU9D and most PedsQL emotional items in infants aged 1–12 months (Table S8) but much stronger correlations for those aged 13–24 months (Table S9). One explanation is that it may be difficult for parents with newborns to identify if the child is annoyed using the CHU9D whereas PedsQL items in EF describe more observable behaviours (e.g. crying or fussing when left along, crying a lot).

Our results have important implications on the choice of instrument in ARM and HD for clinicians and on measuring HRQOL in childhood for instrument developers. PedsQL has been validated for use in children affected by ARM and HD, and CHU9D demonstrated comparable psychometric properties to PedsQL in affected children aged 2 years and above in Australia, suggesting CHU9D is a valid measure of HRQOL in this condition and corresponding age groups. We present the utilities for ARM and HD (ESM Table S12) in children aged 2 years and above, which can be used as reference bases in economic evaluations of targeted interventions. Though both instruments showed comparable performance, the differences in the descriptive systems, including dimensions covered, number of items and recall period, could still lead to differences in the prevalence and distribution of HRQOL problems and different conclusions about the impact of the condition on HRQOL. PedsQL has age-specific versions which were developed to measure aspects of HRQOL most relevant to the corresponding ages. Specifically, items on ‘vomiting’, ‘having gas’, ‘split up after eating’ may be particularly relevant to colorectal conditions whereas an eating-related dimension is not covered in CHU9D. However, the PedsQL has 36 and 45 items for infants which could be a response burden for parents. It also has no preference weights, which limits its use as a measure of HRQOL for economic evaluation. On the other hand, CHU9D is accompanied by preference weights, but it may not be appropriate for use in children under 2 years. It has a version for children under 5 years with guidance notes to parents on how to respond to schoolwork, daily routine and activities dimensions. Though during data collection, we asked parents to think about their child’s abilities with learning tasks and activities as appropriate for their age, we did not use the guidance notes, which limited our validation of the CHU9D adapted version in this age group. Nevertheless, our findings on the weak correlation between its emotional dimensions (e.g. annoyed) and PedsQL EF domain items may suggest that further development (e.g. adding observable signs and symptoms as example for proxy report) could be considered when using CHU9D in very young children [64, 65].

Literature has discussed the challenges in measuring HRQOL in very young children [65, 66] and measuring and valuing HRQOL over the life span. Most existing child HRQOL instruments were developed for older children with adapted versions or guidance for younger children, e.g. EQ-5D-Y-3L [43], with a few instruments designed specific for infants (e.g. IQI [67], EQ-TIPS [65]). If a condition is only relevant to certain ages in childhood or use of the instrument only involves monitoring health for a certain period, it may be less of an issue in terms of choosing the most appropriate instrument. However, if a condition has long-term impact from early years to adolescence or even adulthood, the consistency of measuring and valuing health over the life course needs to be considered. In the case of congenital colorectal conditions, children and families need ongoing care until the transition to adulthood and beyond. Multidisciplinary services have been established in several children’s hospitals worldwide [68], aiming to provide coordinated and transitional care for children and families. Health economic evidence is important to evaluate the benefit of such services and support long-term implementation. Measuring and valuing health long-term is an essential component of the evidence. Except for the EQ-5D-Y, which is a child friendly version developed from its adult HRQOL instrument EQ-5D, existing child HRQOL measures do not have a comparable adult version. This lack of comparable measures may lead to inconsistent measurement of HRQOL over the lifespan. The longitudinal ColoQol study provides a unique opportunity to explore the issues of measuring HRQOL over the life span in colorectal conditions and could provide several points of reference for future interventions. In our ongoing work, we will continue to collect data, and explore validity of alternative instruments in ARM and HD across different ages, for example, EQ-5D-Y-3L, which is adapted for 2–4 years old [66] and EQ-TIPS for infants 1–36 months [65].

Overall, we present the first study on the validity of CHU9D compared to PedsQL in children with ARM or HD. Our study benefits from using a relatively large sample of a rare paediatric condition and the inclusion of infants, toddlers, and children. This enabled the assessment of HRQOL across different ages and stages that can be used for long-term assessment of health outcomes and provide a reference base for potential treatment and interventions. However, there are also several limitations. First, we used the standard proxy report version of CHU9D without guidance notes on schoolwork, daily routine and activities dimension for use in children under 5 years. With guidance notes, we might have fewer missing values for the learning dimension in 2–4 year olds but not necessarily for 1–24 month olds. Second, in our survey PedsQL and CHU9D were asked in a fixed order, and we used interviewer-administered mode via telephone, there may be ordering effects and social desirability bias that may impact on parents’ response [69–71]. Third, despite use of a relatively large sample, the sample size was still inadequate for known-group validation within each condition. Given the ColoQol study is ongoing with further waves, we can explore known-group validity when more data is available. Fourth, all children and families were recruited post-surgery, which is common in clinical cohorts. We were not able to find appropriate anchors to conduct test-retest and responsiveness test. The current survey runs annually, which may be hard to capture timely change in health. Future studies should explore test-retest and responsiveness of instruments.

## Conclusion

This study provides new evidence on the psychometric performance of CHU9D compared to PedsQL in Australian children with ARM and HD. The CHU9D and PedsQL demonstrated comparable and acceptable psychometric properties in children aged 2 years and above. Several PedsQL items for infants aged under 2 years, such as ‘vomiting’, ‘having gas’, ‘split up after eating’ may be particularly relevant to colorectal conditions, but the large item count could be a response burden for parents. The performance of CHU9D in children under 2 years warrants further exploration and modification may be needed for use in this age group, particularly on schoolwork and emotion-related dimensions.

## Electronic supplementary material

Below is the link to the electronic supplementary material.

Supplementary Material 1

## Data Availability

The datasets generated during and/or analysed during the current study are available from the corresponding author on reasonable request.
