# Supplementary material for: Psychometric comparison of CHU9D and PedsQL 4.0 proxy version administered to parents of children with congenital colorectal conditions in Australia
Source: Eur J Health Econ. 2025 Jun 5;26(9):1671–82. doi: 10.1007/s10198-025-01797-0 (PMC12618297; doi:10.1007/s10198-025-01797-0)
Supplement: Supplementary file 1 — Supplementary Material 1 [file 10198_2025_1797_MOESM1_ESM.docx]

**Supplementary materials**

| Table S1. Completion rate of different instrument | | | | | | |
| --- | --- | --- | --- | --- | --- | --- |
| **Instrument** | **All** | **ARM** | **HD** | **1-24 m** | **2-4 years** | **5-7 years** |
| **CHU-9D (N=145)** |  |  |  |  |  |  |
| Worried | 100.0% | 100.0% | 100.0% | 100.0% | 100.0% | 100.0% |
| Sad | 100.0% | 100.0% | 100.0% | 100.0% | 100.0% | 100.0% |
| In pain | 100.0% | 100.0% | 100.0% | 100.0% | 100.0% | 100.0% |
| Tired | 100.0% | 100.0% | 100.0% | 100.0% | 100.0% | 100.0% |
| Annoyed | 100.0% | 100.0% | 100.0% | 100.0% | 100.0% | 100.0% |
| Schoolwork/homework | 86.9% | 87.5% | 86.0% | 76.9% | 87.9% | 100.0% |
| Sleep | 100.0% | 100.0% | 100.0% | 100.0% | 100.0% | 100.0% |
| Daily routine | 100.0% | 100.0% | 100.0% | 100.0% | 100.0% | 100.0% |
| Activities | 100.0% | 100.0% | 100.0% | 100.0% | 100.0% | 100.0% |
| PedsQL Infant 1-24 months (N=52) |  | N=35 | N=17 |  |  |  |
| PF | 100.0% | 100.0% | 100.0% | 100.0% | - | - |
| PS | 100.0% | 100.0% | 100.0% | 100.0% | - | - |
| EF | 100.0% | 100.0% | 100.0% | 100.0% | - | - |
| SF | 100.0% | 100.0% | 100.0% | 100.0% | - | - |
| CF | 100.0% | 100.0% | 100.0% | 100.0% | - | - |
| PedsQL 2-4 years (N=58) |  | N=37 | N=21 |  |  |  |
| PF | 100.0% | 100.0% | 100.0% | - | 100.0% | - |
| EF | 100.0% | 100.0% | 100.0% | - | 100.0% | - |
| SF | 100.0% | 100.0% | 100.0% | - | 100.0% | - |
| SchF | 79.3% | 78.4% | 81.0% | - | 79.3% | - |
| PedsQL 5-7 years (N=35) |  | N=16 | N=19 |  |  |  |
| PF | 100.0% | 100.0% | 100.0% | - | - | 100.0% |
| EF | 100.0% | 100.0% | 100.0% | - | - | 100.0% |
| SF | 100.0% | 100.0% | 100.0% | - | - | 100.0% |
| SchF | 97.1% | 93.8% | 100.0% | - | - | 97.1% |
| ARM: anorectal malformations; HD: Hirschsprung disease. PF: physical functioning; PS: physical symptoms; EF: emotional functioning; SF: social-functioning ; CF: cognitive-functioning; SchF: School functioning. | | | | | | |

| Table S2 CHU9D response distribution by conditions | | | | | | | | | | | |
| --- | --- | --- | --- | --- | --- | --- | --- | --- | --- | --- | --- |
|  | ARM | | | | | HD | | | | |  |
| CHU9D items | Level 1 | Level 2 | Level 3 | Level 4 | Level 5 | Level 1 | Level 2 | Level 3 | Level 4 | Level 5 | p-value |
| 1. Worried | 78.4 | 14.8 | 4.5 | 1.1 | 1.1 | 71.9 | 17.5 | 8.8 | 1.8 | 0.0 | 0.734 |
| 2. Sad | 61.4 | 29.5 | 6.8 | 2.3 | 0.0 | 61.4 | 24.6 | 10.5 | 3.5 | 0.0 | 0.756 |
| 3. In pain | 69.3 | 20.5 | 9.1 | 1.1 | 0.0 | 71.9 | 17.5 | 8.8 | 1.8 | 0.0 | 0.981 |
| 4. Tired | 38.6 | 38.6 | 13.6 | 8.0 | 1.1 | 40.4 | 26.3 | 28.1 | 3.5 | 0.0 | 0.137 |
| 5. Annoyed | 39.8 | 38.6 | 10.2 | 10.2 | 1.1 | 40.4 | 28.1 | 17.5 | 12.3 | 1.8 | 0.558 |
| 6. Schoolwork | 79.2 | 11.7 | 3.9 | 1.3 | 3.9 | 67.3 | 10.2 | 12.2 | 2.0 | 8.2 | 0.279 |
| 7. Sleep | 62.5 | 20.5 | 12.5 | 4.5 | 0.0 | 50.9 | 15.8 | 15.8 | 15.8 | 1.8 | 0.084 |
| 8. Daily routine | 70.5 | 14.8 | 9.1 | 4.5 | 1.1 | 61.4 | 17.5 | 7.0 | 10.5 | 3.5 | 0.461 |
| 9. Activities | 80.7 | 10.2 | 5.7 | 3.4 | 0.0 | 71.9 | 14.0 | 8.8 | 5.3 | 0.0 | 0.655 |
| ARM: anorectal malformations; HD: Hirschsprung disease.  Statistical test: Fisher’s exact test | | | | | | | | | | | |

| Table S3 CHU9D response distribution by age group | | | | | | | | | | | | | | | | |
| --- | --- | --- | --- | --- | --- | --- | --- | --- | --- | --- | --- | --- | --- | --- | --- | --- |
|  | 1-24 months | | | | | 2-4 years | | | | | 5-7 years | | | | |  |
|  | Level 1 | Level 2 | Level 3 | Level 4 | Level 5 | Level 1 | Level 2 | Level 3 | Level 4 | Level 5 | Level 1 | Level 2 | Level 3 | Level 4 | Level 5 | p-value |
| 1. Worried | 84.6 | 11.5 | 1.9 | 1.9 | 0.0 | 75.9 | 15.5 | 6.9 | 0.0 | 1.7 | 62.9 | 22.9 | 11.4 | 2.9 | 0.0 | 0.206 |
| 2. Sad | 63.5 | 25.0 | 9.6 | 1.9 | 0.0 | 58.6 | 29.3 | 8.6 | 3.4 | 0.0 | 62.9 | 28.6 | 5.7 | 2.9 | 0.0 | 0.991 |
| 3. In pain | 61.5 | 21.2 | 17.3 | 0.0 | 0.0 | 74.1 | 19.0 | 6.9 | 0.0 | 0.0 | 77.1 | 17.1 | 0.0 | 5.7 | 0.0 | 0.029 |
| 4. Tired | 28.8 | 38.5 | 25.0 | 5.8 | 1.9 | 44.8 | 31.0 | 15.5 | 6.9 | 1.7 | 45.7 | 31.4 | 17.1 | 5.7 | 0.0 | 0.758 |
| 5. Annoyed | 44.2 | 32.7 | 15.4 | 5.8 | 1.9 | 43.1 | 36.2 | 10.3 | 8.6 | 1.7 | 28.6 | 34.3 | 14.3 | 22.9 | 0.0 | 0.366 |
| 6. Schoolwork | 87.5 | 0.0 | 5.0 | 2.5 | 5.0 | 80.4 | 0.0 | 11.8 | 3.9 | 3.9 | 51.4 | 22.9 | 14.3 | 2.9 | 8.6 | 0.003 |
| 7. Sleep | 61.5 | 17.3 | 19.2 | 1.9 | 0.0 | 55.2 | 20.7 | 10.3 | 12.1 | 1.7 | 57.1 | 17.1 | 11.4 | 14.3 | 0.0 | 0.330 |
| 8. Daily routine | 67.3 | 17.3 | 7.7 | 3.8 | 3.8 | 72.4 | 15.5 | 6.9 | 5.2 | 0.0 | 57.1 | 14.3 | 11.4 | 14.3 | 2.9 | 0.516 |
| 9. Activities | 88.5 | 5.8 | 3.8 | 1.9 | 0.0 | 82.8 | 10.3 | 5.2 | 1.7 | 0.0 | 51.4 | 22.9 | 14.3 | 11.4 | 0.0 | 0.004 |
| ARM: anorectal malformations; HD: Hirschsprung disease.  Statistical test: Fisher’s exact test | | | | | | | | | | | | | | | | |

| Table S4 Known group validity of CHU9D and PedsQL for different groups | | | | | | | | | | | |
| --- | --- | --- | --- | --- | --- | --- | --- | --- | --- | --- | --- |
| Groups | Sample size | PedsQl total scores | | | | | CHU9D utilities (UK value set) | | | | |
|  |  | mean | SD | diff | p-value | Cohen's d ES | mean | SD | diff | p-value | Cohen's d ES |
| Clinical conditions | |  |  |  |  |  |  |  |  |  |  |
| ARM | 77 | 79.40 | 14.18 | -4.21 | 0.206 | 0.27 | 0.87 | 0.09 | -0.03 | 0.333 | 0.25 |
| HD | 49 | 75.19 | 17.35 |  |  |  | 0.85 | 0.12 |  |  |  |
| ARM type |  |  |  |  |  |  |  |  |  |  |  |
| Simple | 56 | 79.09 | 13.97 | 1.14 | 0.572 | 0.08 | 0.88 | 0.09 | -0.01 | 0.619 | 0.07 |
| Complex | 21 | 80.23 | 15.05 |  |  |  | 0.87 | 0.09 |  |  |  |
| HD segment |  |  |  |  |  |  |  |  |  |  |  |
| Short | 15 | 70.60 | 18.85 | 6.61 | 0.242 | 0.37 | 0.84 | 0.10 | 0.01 | 0.640 | 0.08 |
| Other | 34 | 77.22 | 16.54 |  |  |  | 0.85 | 0.13 |  |  |  |
| Age group |  |  |  |  |  |  |  |  |  |  |  |
| 1-24 month(ref) | 40 | 76.41 | 12.29 |  | 0.093 |  | 0.88 | 0.10 |  | 0.180 |  |
| 2-4 years | 51 | 81.30 | 14.87 | 4.89 |  | 0.36 | 0.87 | 0.09 | -0.01 |  | 0.06 |
| 5-7 years | 35 | 74.17 | 18.91 | -2.24 |  | 0.14 | 0.83 | 0.12 | -0.05 |  | 0.42 |
| Standard thresholds 0.2 to < 0.5, 0.5 to < 0.8, and 0.8 or more denote small, medium, and large effect sizes, respectively. Known group validity: n≥100 per group very good; n=50-99 per group adequate; n=30-49 per group doubtful; n<30 per group inadequate.  ARM: anorectal malformations; HD: Hirschsprung disease. SD: standard deviation | | | | | | | | | | | |

| Table S5 Known group validity of CHU9D and PedsQL dimensions for different groups, by condition and age | | | | | | | | | | | | | | | | | | | | | |
| --- | --- | --- | --- | --- | --- | --- | --- | --- | --- | --- | --- | --- | --- | --- | --- | --- | --- | --- | --- | --- | --- |
|  | ARM | | | HD | | |  |  | 1-24 months | | | 2-4 years | | | 5-7 years | | |  |  | | |
|  | N | mean | SD | N | mean | SD | p-value | Cohen's d ES | N | mean | SD | N | mean | SD | N | mean | SD | p-value | Cohen's d ES | | |
| **CHU9D** |  |  |  |  |  |  |  |  |  |  |  |  |  |  |  |  |  |  | 2-4 | | 5-7 |
| Worried | 77 | 1.31 | 0.73 | 49 | 1.43 | 0.76 | 0.301 | 0.16 | 40 | 1.15 | 0.53 | 51 | 1.39 | 0.80 | 35 | 1.54 | 0.82 | **0.022** | 0.36 | 0.57 | |
| Sad | 77 | 1.51 | 0.74 | 49 | 1.55 | 0.82 | 0.916 | 0.06 | 40 | 1.50 | 0.75 | 51 | 1.57 | 0.81 | 35 | 1.49 | 0.74 | 0.886 | 0.09 | 0.02 | |
| Pain | 77 | 1.43 | 0.70 | 49 | 1.41 | 0.73 | 0.745 | 0.03 | 40 | 1.55 | 0.75 | 51 | 1.37 | 0.63 | 35 | 1.34 | 0.76 | 0.247 | 0.26 | 0.27 | |
| Tired | 77 | 1.92 | 0.97 | 49 | 1.96 | 1.02 | 0.872 | 0.04 | 40 | 2.08 | 1.02 | 51 | 1.90 | 1.01 | 35 | 1.83 | 0.92 | 0.512 | 0.17 | 0.25 | |
| Annoyed | 77 | 1.96 | 1.03 | 49 | 2.10 | 1.14 | 0.573 | 0.13 | 40 | 1.83 | 1.01 | 51 | 1.96 | 1.06 | 35 | 2.31 | 1.13 | 0.121 | 0.13 | 0.46 | |
| School | 77 | 1.39 | 0.93 | 49 | 1.73 | 1.25 | 0.101 | 0.31 | 40 | 1.38 | 1.05 | 51 | 1.35 | 0.89 | 35 | 1.94 | 1.26 | **0.002** | 0.02 | 0.49 | |
| Sleep | 77 | 1.64 | 0.92 | 49 | 2.14 | 1.26 | **0.030** | 0.46 | 40 | 1.75 | 0.93 | 51 | 1.90 | 1.19 | 35 | 1.83 | 1.12 | 0.939 | 0.14 | 0.08 | |
| Daily routine | 77 | 1.56 | 0.97 | 49 | 1.86 | 1.24 | 0.200 | 0.27 | 40 | 1.70 | 1.16 | 51 | 1.49 | 0.88 | 35 | 1.91 | 1.25 | 0.302 | 0.20 | 0.18 | |
| Activities | 77 | 1.32 | 0.75 | 49 | 1.53 | 0.92 | 0.157 | 0.25 | 40 | 1.15 | 0.58 | 51 | 1.29 | 0.67 | 35 | 1.86 | 1.06 | **0.000** | 0.23 | 0.83 | |
| **PedsQL** |  |  |  |  |  |  |  |  |  |  |  |  |  |  |  |  |  |  |  |  | |
| PF | 77 | 79.78 | 19.52 | 49 | 79.33 | 20.05 | 0.921 | 0.02 | 40 | 76.08 | 17.93 | 51 | 83.09 | 18.33 | 35 | 78.57 | 22.87 | 0.128 | 0.39 | 0.12 | |
| PS | 28 | 77.05 | 13.23 | 12 | 77.71 | 12.31 | 0.959 | 0.05 | 40 | 77.25 | 12.81 | - | - | - | - | - | - | - | - | - | |
| EF | 77 | 71.98 | 15.21 | 49 | 66.99 | 20.20 | 0.244 | 0.28 | 40 | 67.50 | 15.39 | 51 | 73.82 | 17.02 | 35 | 67.43 | 19.53 | 0.196 | 0.39 | 0.00 | |
| SF | 77 | 87.66 | 15.96 | 49 | 81.22 | 21.82 | 0.122 | 0.34 | 40 | 90.25 | 12.57 | 51 | 87.16 | 18.12 | 35 | 76.43 | 22.35 | **0.004** | 0.20 | 0.76 | |
| CF | 28 | 83.43 | 22.68 | 12 | 81.02 | 15.17 | 0.358 | 0.13 | 40 | 82.71 | 20.55 | - | - | - | - | - | - | - | - | - | |
| School | 42 | 77.62 | 21.20 | 34 | 70.56 | 23.43 | 0.188 | 0.32 | - | - | - | 42 | 77.18 | 22.17 | 34 | 71.10 | 22.46 | 0.147 |  |  | |
| Standard thresholds 0.2 to < 0.5, 0.5 to < 0.8, and 0.8 or more denote small, medium, and large effect sizes, respectively. Known group validity: n≥100 per group very good; n=50-99 per group adequate; n=30-49 per group doubtful; n<30 per group inadequate. The reference group in age group was 1-24 months.  SD: standard deviation. ARM: anorectal malformations; HD: Hirschsprung disease.  PF: physical functioning ; PS: physical symptoms; EF: emotional functioning; SF: social-functioning: CF: cognitive-functioning. School: School functioning | | | | | | | | | | | | | | | | | | | | | |

| Table S6 Known group validity of CHU9D and PedsQL dimensions for different groups by type within conditions | | | | | | | | | | | | | | | | |
| --- | --- | --- | --- | --- | --- | --- | --- | --- | --- | --- | --- | --- | --- | --- | --- | --- |
| Groups | ARM simple | | | ARM complex | | |  |  | HD other | | | HD short | | |  |  |
|  | N | mean | SD | N | mean | SD | p-value | Cohen's d ES | N | mean | SD | N | mean | SD | p-value | Cohen's d ES |
| CHU9D |  |  |  |  |  |  |  |  |  |  |  |  |  |  |  |  |
| Worried | 56 | 1.32 | 0.79 | 21 | 1.29 | 0.56 | 0.862 | 0.05 | 15 | 1.40 | 0.63 | 34 | 1.44 | 0.82 | 0.861 | 0.06 |
| Sad | 56 | 1.50 | 0.76 | 21 | 1.52 | 0.68 | 0.759 | 0.03 | 15 | 1.47 | 0.92 | 34 | 1.59 | 0.78 | 0.380 | 0.14 |
| Pain | 56 | 1.46 | 0.74 | 21 | 1.33 | 0.58 | 0.630 | 0.20 | 15 | 1.53 | 0.74 | 34 | 1.35 | 0.73 | 0.338 | 0.24 |
| Tired | 56 | 1.89 | 1.02 | 21 | 2.00 | 0.84 | 0.391 | 0.11 | 15 | 2.40 | 1.18 | 34 | 1.76 | 0.89 | 0.071 | 0.61 |
| Annoyed | 56 | 1.95 | 1.03 | 21 | 2.00 | 1.05 | 0.836 | 0.05 | 15 | 2.13 | 1.30 | 34 | 2.09 | 1.08 | 0.978 | 0.04 |
| School | 56 | 1.43 | 0.95 | 21 | 1.29 | 0.90 | 0.455 | 0.15 | 15 | 1.47 | 0.83 | 34 | 1.85 | 1.40 | 0.462 | 0.34 |
| Sleep | 56 | 1.64 | 0.86 | 21 | 1.62 | 1.07 | 0.656 | 0.02 | 15 | 2.73 | 1.10 | 34 | 1.88 | 1.25 | 0.022 | 0.72 |
| Daily routine | 56 | 1.54 | 0.97 | 21 | 1.62 | 0.97 | 0.539 | 0.09 | 15 | 1.60 | 0.74 | 34 | 1.97 | 1.40 | 0.848 | 0.33 |
| Activities | 56 | 1.36 | 0.82 | 21 | 1.24 | 0.54 | 0.833 | 0.17 | 15 | 1.60 | 0.99 | 34 | 1.50 | 0.90 | 0.786 | 0.11 |
| PedsQL |  |  |  |  |  |  |  |  |  |  |  |  |  |  |  |  |
| PF | 56 | 79.32 | 19.75 | 21 | 81.04 | 19.31 | 0.774 | 0.09 | 15 | 74.63 | 22.16 | 34 | 81.40 | 19.03 | 0.244 | 0.33 |
| PS | 22 | 77.16 | 13.74 | 6 | 76.67 | 12.32 | 0.990 | 0.04 | 4 | 69.38 | 15.19 | 8 | 81.88 | 8.94 | 0.202 | 1.00 |
| EF | 56 | 71.15 | 15.81 | 21 | 74.19 | 13.62 | 0.401 | 0.21 | 15 | 63.36 | 20.93 | 34 | 68.59 | 19.97 | 0.357 | 0.26 |
| SF | 56 | 87.14 | 15.74 | 21 | 89.05 | 16.85 | 0.378 | 0.12 | 15 | 77.67 | 20.67 | 34 | 82.79 | 22.43 | 0.344 | 0.24 |
| CF | 22 | 81.00 | 24.05 | 6 | 92.36 | 15.00 | 0.190 | 0.57 | 4 | 79.69 | 15.63 | 8 | 81.68 | 15.98 | 0.836 | 0.13 |
| School | 31 | 81.24 | 17.49 | 11 | 67.42 | 27.77 | 0.129 | 0.60 | 9 | 58.52 | 25.97 | 25 | 74.90 | 21.35 | 0.087 | 0.69 |
| Standard thresholds 0.2 to < 0.5, 0.5 to < 0.8, and 0.8 or more denote small, medium, and large effect sizes, respectively. Known group validity: n≥100 per group very good; n=50-99 per group adequate; n=30-49 per group doubtful; n<30 per group inadequate.  SD: standard deviation. ARM: anorectal malformations; HD: Hirschsprung disease.  PF: physical functioning ; PS: physical symptoms; EF: emotional functioning; SF: social-functioning: CF: cognitive-functioning. School: School functioning | | | | | | | | | | | | | | | | |

| Table S7 Known group validity of CHU9D and PedsQL for colorectal conditions compared with healthy controls | | | | | | | | | | | |
| --- | --- | --- | --- | --- | --- | --- | --- | --- | --- | --- | --- |
| Groups | Sample size | PedsQl total scores | | | | | CHU9D utilities | | | | |
|  |  | mean | SD | diff | p-value | Cohen's d ES | mean | SD | diff | p-value | Cohen's d ES |
| Healthy controls (2-4 years old) | 267 | 84.25 | 14.79 |  | NA |  |  |  |  | NA |  |
| 2-4 years old |  |  |  |  |  |  |  |  |  |  |  |
| ARM | 33 | 82.88 | 13.24 | -1.37 |  | 0.10 | 0.79 | 0.16 | -0.05 |  | 0.32 |
| HD | 18 | 78.40 | 17.51 | -5.85 |  | 0.36 | 0.66 | 0.23 | -0.18 |  | 0.90 |
| 2-7 years old |  |  |  |  | NA |  |  |  |  | NA |  |
| ARM | 49 | 81.18 | 14.61 | -3.07 |  | 0.21 | 0.75 | 0.17 | -0.09 |  | 0.53 |
| HD | 37 | 74.71 | 19.11 | -9.54 |  | 0.56 | 0.67 | 0.25 | -0.17 |  | 0.81 |
| Standard thresholds 0.2 to < 0.5, 0.5 to < 0.8, and 0.8 or more denote small, medium, and large effect sizes, respectively. Known group validity: n≥100 per group very good; n=50-99 per group adequate; n=30-49 per group doubtful; n<30 per group inadequate.  ARM: anorectal malformations; HD: Hirschsprung disease. SD: standard deviation  Data for healthy controls were obtained from Xiong, X., et al., Psychometric Properties of Child Health Utility 9D (CHU9D) Proxy Version Administered to Parents and Caregivers of Children Aged 2–4 Years Compared with Pediatric Quality of Life Inventory™ (PedsQL). PharmacoEconomics, 2024. 42(1): p. 147-161. | | | | | | | | | | | |

| Table S8 Convergence between CHU9D dimensions and PedsQL items in children aged 1-12 months | | | | | | | | | | |
| --- | --- | --- | --- | --- | --- | --- | --- | --- | --- | --- |
| PedsQL （1-12 months) | | **CHU9D** | | | | | | | | |
|  |  | Worried | Sad | Pain | Tired | Annoyed | School | Sleep | Daily routine | Activities |
| PF | 1. Low energy level | -0.2607 | 0.0902 | **-0.3850*** | 0.2263 | 0.2181 | -0.0218 | -0.0439 | **-0.3094** | -0.2511 |
|  | 2. Difficulty participating in active play | **-0.3143** | 0.0554 | -0.1374 | 0.2713 | 0.2044 | 0.2323 | **-0.3366** | -0.1817 | -0.2804 |
|  | 3. Having hurts or aches | **-0.3647*** | -0.2598 | **-0.4085*** | -0.2996 | **-0.3259** | -0.0217 | **-0.334** | -0.1838 | -0.0643 |
|  | 4. Feeling tired | **-0.4254*** | **-0.4676*** | **-0.4015*** | **-0.5130*** | **-0.3831*** | 0.0961 | -0.2542 | -0.0709 | -0.2866 |
|  | 5. Being lethargic | -0.1373 | **-0.3696*** | -0.1953 | -0.0232 | -0.2558 | -0.0329 | -0.13 | -0.2424 | -0.2257 |
|  | 6. Resting a lot | -0.1631 | -0.1793 | **-0.3155** | -0.0635 | **-0.3048** | 0.0636 | 0.1154 | -0.0748 | **-0.3341** |
| PS | 1. Having gas | 0.0617 | -0.1661 | -0.2049 | -0.2758 | -0.2597 | **-0.3229** | **-0.3459*** | -0.2753 | 0.1317 |
|  | 2. Spitting up after eating | 0.043 | 0.2982 | -0.2532 | -0.0121 | 0.1839 | 0.0215 | -0.0423 | -0.1254 | -0.0599 |
|  | 3. Difficulty breathing | -0.0068 | -0.1658 | -0.2539 | -0.0849 | 0.1141 | 0.1469 | -0.1972 | -0.1021 | 0.1564 |
|  | 4. Being sick to his/her stomach | -0.1435 | 0.0645 | 0.2084 | 0.1851 | 0.0812 | -0.0873 | -0.0772 | -0.1613 | 0.2573 |
|  | 5. Difficulty swallowing | 0.1313 | 0.0159 | 0.1291 | 0.0891 | 0.0563 | -0.0481 | 0.0112 | -0.2201 | 0.2245 |
|  | 6. Being constipated | -0.1603 | -0.0198 | **-0.4507*** | -0.2069 | **-0.3853*** | **-0.4023** | **-0.4161*** | -0.2498 | -0.0108 |
|  | 7. Having a rash | -0.1084 | -0.1176 | 0.055 | -0.0767 | -0.096 | 0.0539 | **-0.3697*** | -0.2064 | 0.1014 |
|  | 8. Having diarrhea | 0.0953 | 0.0607 | 0.1271 | -0.0074 | -0.2161 | 0.1915 | -0.006 | -0.1269 | 0.1301 |
|  | 9. Wheezing | -0.0004 | -0.0729 | 0.0975 | 0.2574 | 0.048 | 0.1466 | -0.097 | -0.2697 | 0.1562 |
|  | 10.Vomiting | 0.0507 | 0.2113 | 0.036 | 0.2815 | **0.3378** | -0.1576 | -0.0069 | -0.2566 | 0.1575 |
| EF | 1. Feeling afraid or scared | -0.1271 | -0.2502 | -0.2811 | -0.0548 | -0.2451 | 0.0771 | -0.17 | -0.2869 | -0.0427 |
|  | 2. Feeling angry | -0.0356 | 0.1294 | -0.0254 | 0.1073 | -0.2341 | 0.1626 | 0.1176 | 0.0632 | 0.0691 |
|  | 3. Crying or fussing when left alone | 0.1213 | 0.0627 | -0.0284 | -0.0763 | **-0.312** | 0.0876 | -0.0504 | -0.0826 | 0.0475 |
|  | 4. Difficulty soothing himself/herself when upset | -0.1503 | -0.0185 | -0.1451 | **-0.3439** | -0.0698 | 0.1774 | -0.2747 | -0.1429 | -0.0351 |
|  | 5. Difficulty falling asleep | **-0.3203** | 0.0834 | -0.0386 | -0.2512 | -0.0265 | -0.0866 | **-0.4743*** | **-0.3512*** | -0.0676 |
|  | 6. Crying or fussing while being cuddled | -0.1523 | 0.0674 | -0.0743 | -0.2937 | -0.2992 | -0.0111 | -0.1403 | -0.1001 | 0.0579 |
|  | 7. Feeling sad | **-0.3567*** | **-0.3660*** | -0.11 | -0.1124 | -0.2857 | -0.2747 | -0.1394 | **-0.3042** | -0.0762 |
|  | 8. Difficulty being soothed when picked up or held | -0.0221 | -0.1368 | 0.0715 | **-0.3099** | -0.2568 | -0.0328 | -0.156 | 0.0567 | 0.2212 |
|  | 9. Difficulty sleeping mostly through the night | -0.0765 | 0.0703 | -0.0271 | -0.2141 | 0.04 | **-0.3891** | **-0.4588*** | **-0.3403** | 0.2308 |
|  | 10. Crying a lot | -0.2717 | -0.1723 | **-0.3556*** | -0.2494 | -0.1356 | -0.1099 | -0.007 | -0.1601 | -0.0334 |
|  | 11. Feeling cranky | -0.1965 | -0.1093 | -0.1333 | **-0.3422** | **-0.313** | **-0.3465** | -0.2447 | -0.2014 | 0.006 |
|  | 12. Difficulty taking naps during the day | -0.2873 | 0.1552 | -0.241 | **-0.339** | -0.0972 | **-0.3178** | **-0.4854*** | -0.1811 | -0.154 |
| SF | 1. Not smiling at others | -0.2729 | 0.0597 | 0.0479 | -0.0943 | -0.1086 | 0.2325 | **-0.311** | 0.0335 | **-0.3235** |
|  | 2. Not laughing when tickled | -0.1646 | 0.2453 | 0.0732 | 0.2262 | 0.015 | 0.2006 | -0.2403 | 0.1127 | -0.1069 |
|  | 3. Not making eye contact with a caregiver | -0.1626 | 0.1924 | 0.0467 | 0.1799 | -0.2386 | 0.1287 | -0.2662 | -0.01 | -0.2474 |
|  | 4. Not laughing when cuddled | **-0.3596** | 0.1304 | 0.0809 | 0.1084 | 0.0295 | 0.1988 | -0.2255 | 0.1286 | -0.0911 |
| CF | 1. Not imitating caregivers' actions | -0.0669 | 0.1134 | -0.0372 | 0.0616 | 0.0633 | 0.0667 | -0.0697 | 0.2783 | -0.082 |
|  | 2. Not imitating caregivers' facial expressions | **-0.3055** | -0.0428 | 0.1106 | 0.1276 | 0.0348 | 0.2692 | -0.1066 | **0.3123** | -0.0709 |
|  | 3. Not imitating caregivers' sounds | -0.0747 | -0.1014 | -0.0305 | 0.1235 | -0.1254 | -0.11 | -0.2548 | 0.0984 | -0.1109 |
|  | 4. Not able to fix his/her attention on objects | -0.1261 | 0.1768 | -0.1516 | 0.1348 | -0.045 | -0.1439 | **-0.4266*** | -0.127 | -0.1022 |
| Correlation coefficients were calculated by Spearman rank correlation. *: correlation significant at 0.05 level..  High correlations, ≥ 0.5 (green); moderate correlations, 0.3–0.49 (orange); low correlation, < 0.3 (black).  Grey shaded cell indicates expected moderate or high correlations (r ≥ 0.3) based on highly similar items in line with published technical guide  PF: physical functioning ; PS: physical symptoms; EF: emotional functioning; SF: social-functioning: CF: cognitive-functioning. | | | | | | | | | | |

| Table S9 Convergence between CHU9D dimensions and PedsQL items in children aged 13-24 months | | | | | | | | | | |
| --- | --- | --- | --- | --- | --- | --- | --- | --- | --- | --- |
| PedsQL （13- 24 months) | | CHU9D | | | | | | | | |
|  |  | Worried | Sad | Pain | Tired | Annoyed | School | Sleep | Daily routine | Activities |
| PF | 1. Low energy level | **-0.3195** | **-0.4952*** | -0.0246 | -0.0263 | -0.1248 | **-0.4734*** | -0.2481 | **-0.5905*** | -0.0336 |
|  | 2. Difficulty participating in active play | **-0.4049** | **-0.4381** | -0.0403 | **-0.3186** | -0.297 | **-0.3922** | **-0.3311** | -0.0861 | **-0.5768*** |
|  | 3. Having hurts or aches | -0.1726 | -0.1272 | -0.0975 | 0.048 | **-0.4837** | -0.0679 | **-0.6600*** | -0.2194 | **-0.3945** |
|  | 4. Feeling tired | -0.1387 | -0.2188 | 0.0709 | -0.2006 | -0.1292 | 0.0868 | -0.1276 | -0.0944 | -0.1387 |
|  | 5. Being lethargic | 0.1136 | -0.1067 | 0.2962 | 0.0968 | -0.1215 | -0.0803 | -0.1388 | 0.2417 | -0.0461 |
|  | 6. Resting a lot | **-0.3855** | **-0.5341*** | -0.1102 | **-0.3768** | **-0.5307*** | **-0.4242** | **-0.4495** | -0.0728 | **-0.4985*** |
|  | 7. Feeling too tired to play | 0.0148 | -0.2531 | 0.1565 | -0.162 | -0.2085 | -0.1618 | -0.2123 | 0.133 | -0.1188 |
|  | 8. Difficulty walking | **-0.4803** | **-0.5154*** | -0.0975 | **-0.3438** | **-0.3678** | **-0.4698*** | **-0.3373** | -0.1554 | **-0.6180*** |
|  | 9. Difficulty running a short distance without falling | **-0.3277** | **-0.6453*** | -0.2263 | **-0.3496** | **-0.7217*** | **-0.5615*** | -0.2153 | 0.0544 | **-0.5327*** |
| PS | 1. Having gas | -0.1795 | -0.1827 | 0.0327 | 0.139 | -0.1207 | -0.0615 | **-0.4271** | **-0.6900*** | **-0.3167** |
|  | 2. Spitting up after eating | -0.1235 | -0.167 | 0.215 | 0.2872 | -0.1381 | -0.2831 | 0.1721 | -0.2334 | 0.2022 |
|  | 3. Difficulty breathing | 0.2327 | **-0.3366** | -0.0681 | -0.1336 | **-0.3047** | **-0.4225** | -0.1584 | -0.1506 | -0.1163 |
|  | 4. Being sick to his/her stomach | -0.1804 | -0.2813 | -0.1762 | 0.1168 | -0.1863 | -0.2941 | **-0.6489*** | **-0.5404*** | -0.1804 |
|  | 5. Difficulty swallowing | 0.1756 | **-0.4126** | -0.2233 | -0.1008 | -0.1117 | **-0.4564** | 0.0715 | **-0.3375** | 0.1756 |
|  | 6. Being constipated | -0.0363 | **-0.4516** | **-0.4954*** | **-0.4311** | -0.1635 | **-0.3499** | -0.1752 | -0.2589 | -0.1765 |
|  | 7. Having a rash | 0.0831 | 0.0184 | -0.2458 | 0.1266 | 0.2985 | -0.0445 | -0.148 | 0.0086 | 0.251 |
|  | 8. Having diarrhea | **-0.3374** | **-0.4811*** | -0.2204 | -0.0248 | -0.0637 | **-0.5201*** | -0.2318 | **-0.3953** | -0.1661 |
|  | 9. Wheezing | 0.229 | -0.1345 | -0.2241 | -0.2773 | **-0.3203** | **-0.409** | -0.0205 | -0.075 | -0.0783 |
|  | 10.Vomiting | -0.2047 | **-0.5583*** | -0.2876 | -0.1175 | **-0.4748** | **-0.6403*** | **-0.3464** | **-0.5624*** | -0.2047 |
| EF | 1. Feeling afraid or scared | **-0.4753*** | **-0.5468*** | -0.2474 | **-0.3194** | **-0.4295** | **-0.4849*** | -0.0843 | -0.266 | **-0.4753*** |
|  | 2. Feeling angry | -0.2684 | -0.2124 | -0.2404 | -0.0436 | -0.1969 | **-0.33** | -0.1963 | **-0.4752*** | -0.0618 |
|  | 3. Crying or fussing when left alone | **-0.389** | **-0.4655*** | 0.217 | 0.0694 | **-0.4601** | **-0.3368** | -0.2046 | **-0.5449*** | **-0.389** |
|  | 4. Difficulty soothing himself/herself when upset | **-0.4316** | **-0.6678*** | -0.1374 | -0.2917 | **-0.3658** | **-0.3367** | **-0.427** | -0.2491 | **-0.4316** |
|  | 5. Difficulty falling asleep | **-0.3525** | -0.2899 | -0.2431 | -0.1545 | **-0.7194*** | **-0.4246** | **-0.6619*** | -0.0467 | **-0.3525** |
|  | 6. Crying or fussing while being cuddled | **-0.3591** | **-0.3785** | 0.0697 | **-0.3114** | -0.2215 | **-0.4087** | -0.1886 | -0.1874 | **-0.4952*** |
|  | 7. Feeling sad | **-0.3629** | **-0.3863** | -0.2253 | **-0.353** | **-0.3571** | -0.235 | **-0.3899** | **-0.4111** | **-0.4948*** |
|  | 8. Difficulty being soothed when picked up or held | 0.049 | 0.2411 | 0.0143 | -0.2368 | 0.1885 | -0.0086 | 0.082 | 0.026 | -0.0816 |
|  | 9. Difficulty sleeping mostly through the night | **-0.3812** | -0.0263 | 0.1384 | 0.2534 | -0.2397 | 0.1302 | **-0.4292** | -0.2667 | **-0.3812** |
|  | 10. Crying a lot | **-0.4639*** | **-0.4078** | -0.1863 | -0.0851 | **-0.5167*** | **-0.3743** | **-0.5334*** | **-0.3471** | -0.2931 |
|  | 11. Feeling cranky | **-0.3477** | **-0.5586*** | **-0.3256** | **-0.3467** | **-0.3137** | **-0.3939** | **-0.3587** | **-0.3444** | **-0.3477** |
|  | 12. Difficulty taking naps during the day | 0.0965 | -0.2155 | 0.1429 | -0.0292 | 0.0293 | -0.2577 | 0.0276 | -0.1431 | 0 |
| SF | 1. Not smiling at others | -0.0325 | **-0.6110*** | **-0.5268*** | **-0.5324*** | -0.2244 | **-0.5613*** | -0.0674 | -0.2715 | **-0.3935** |
|  | 2. Not laughing when tickled | **-0.3967** | **-0.3328** | -0.1981 | **-0.3193** | **-0.8171*** | **-0.4803*** | **-0.3269** | **-0.3462** | **-0.8168*** |
|  | 3. Not making eye contact with a caregiver | 0.0807 | -0.2252 | -0.1418 | -0.0463 | **-0.4873** | **-0.3833** | -0.2827 | **-0.3134** | 0.0807 |
|  | 4. Not laughing when cuddled | -0.0135 | 0.0125 | -0.0947 | -0.0425 | -0.2253 | **-0.3576** | -0.1062 | -0.1932 | **-0.3773** |
|  | 5. Being uncomfortable around other children | **-0.6351*** | **-0.5130*** | **-0.4188** | **-0.4499** | -0.5316 | **-0.3948** | -0.2359 | -0.2957 | -0.2887 |
| CF | 1. Not imitating caregivers' actions | **-0.3325** | -0.1961 | -0.0568 | -0.1306 | **-0.6143*** | -0.2573 | **-0.4346** | **-0.4668** | **-0.7273*** |
|  | 2. Not imitating caregivers' facial expressions | -0.2868 | -0.2779 | -0.1101 | -0.1524 | **-0.8054*** | -0.2469 | **-0.5189*** | **-0.3489** | **-0.6882*** |
|  | 3. Not imitating caregivers' sounds | -0.2657 | **-0.3433** | -0.2054 | **-0.3173** | **-0.4832** | **-0.3521** | -0.268 | **-0.3067** | **-0.6866*** |
|  | 4. Not able to fix his/her attention on objects | 0.2303 | 0.0714 | 0.0319 | 0.1183 | -0.1117 | 0.0237 | 0.0601 | **-0.4924*** | -0.097 |
|  | 5. Not imitating caregivers' speech | -0.1807 | **-0.3389** | -0.1004 | **-0.3594** | -0.2947 | -0.2127 | **-0.3083** | -0.2974 | **-0.5955*** |
|  | 6. Difficulty pointing to his/her body parts when asked | -0.2231 | -0.1501 | 0.0402 | -0.0635 | **-0.406** | -0.237 | -0.1742 | -0.2284 | **-0.6178*** |
|  | 7. Difficulty naming familiar objects | **-0.5012** | -0.1591 | 0.1456 | -0.0017 | -0.5062 | -0.2298 | -0.0839 | **-0.3268** | **-0.6450*** |
|  | 8. Difficulty repeating words | **-0.4614** | -0.2677 | 0.062 | -0.0471 | **-0.4285** | **-0.3606** | -0.1157 | -0.2227 | **-0.6159*** |
|  | 9. Difficulty keeping his/her attention on things | **-0.4233** | **-0.5278*** | -0.1206 | -0.1456 | -0.5031 | **-0.6248*** | -0.2025 | **-0.4077** | **-0.4233** |
| Correlation coefficients were calculated by Spearman rank correlation. *: correlation significant at 0.05 level. High correlations, ≥ 0.5 (green); moderate correlations, 0.3–0.49 (orange); low correlation, < 0.3 (black). Grey shaded cell indicates expected moderate or high correlations (r ≥ 0.3) based on highly similar items in line with published technical guide.PF: physical functioning ; PS: physical symptoms; EF: emotional functioning; SF: social-functioning: CF: cognitive-functioning. | | | | | | | | | | |

| Table S10 Convergence between CHU9D dimensions and PedsQL items in children aged 13-24 months | | | | | | | | | | |
| --- | --- | --- | --- | --- | --- | --- | --- | --- | --- | --- |
| PedsQL (2-4 years) | | **CHU9D** | | | | | | | | |
|  |  | Worried | Sad | Pain | Tired | Annoyed | School | Sleep | Daily routine | Activities |
| PF | Walking | -0.2197 | -0.1964 | **-0.3265*** | -0.2535 | -0.1601 | -0.2537 | -0.1598 | -0.1515 | -0.0802 |
|  | Running | -0.1345 | -0.2235 | -0.2699 | -0.2172 | -0.1256 | -0.1483 | -0.1896 | -0.1251 | -0.2164 |
|  | Participating in active play or exercise | -0.1862 | -0.1391 | **-0.3281*** | -0.2688* | -0.0555 | -0.1131 | -0.2004 | -0.1244 | **-0.3439*** |
|  | Lifting something heavy | 0.044 | -0.0558 | -0.2079 | -0.0734 | 0.0174 | -0.214 | -0.1151 | **-0.3238*** | -0.2828* |
|  | Bathing | -0.0496 | -0.1002 | -0.2027 | -0.1239 | -0.1021 | -0.2336 | -0.1968 | **-0.5280*** | -0.2201 |
|  | Helping to pick up his or her toys | -0.1793 | -0.1819 | -0.2419 | **-0.3179*** | -0.2790* | -0.2572 | **-0.2406** | 0.0086 | -0.0141 |
|  | Having aches or pains | -0.0356 | -0.1919 | **-0.4974*** | -0.2036 | **-0.3051*** | 0.1224 | -0.1971 | 0.0302 | 0.0013 |
|  | Having low energy | 0.0347 | -0.0769 | -0.2606 | -0.1863 | **-0.3038*** | 0.0091 | -0.1346 | -0.1074 | -0.0733 |
| EF | Feeling afraid or scared | -0.1668 | -0.1362 | -0.2369 | -0.0464 | -0.2266 | -0.0993 | -0.1762 | -0.2302 | **-0.3228*** |
|  | Feeling sad | -0.2936* | -0.2999* | -0.0251 | -0.1548 | **-0.5268*** | -0.0015 | -0.2363 | -0.1012 | 0.0331 |
|  | Feeling angry | -0.1907 | -0.2308 | 0.0456 | -0.0413 | **-0.4280*** | -0.0652 | -0.1772 | -0.167 | -0.0214 |
|  | Having trouble sleeping | -0.1201 | -0.2293 | -0.2289 | **-0.3662*** | -0.2205 | -0.224 | **-0.6406*** | -0.0366 | -0.0979 |
|  | Worrying | **-0.4788*** | **-0.3435*** | -0.1827 | -0.156 | **-0.3980*** | -0.1497 | **-0.3254*** | -0.1773 | -0.078 |
| SF | Playing with other children | -0.1675 | -0.0573 | -0.0881 | -0.1682 | 0.1244 | **-0.3347*** | **-0.4708*** | **-0.3633*** | -0.2351 |
|  | Other children not wanting to play with him or her | -0.1593 | -0.1329 | -0.073 | -0.0801 | -0.1271 | **-0.4038*** | **-0.3113*** | **-0.4936*** | **-0.3212*** |
|  | Getting teased by other children | -0.1303 | -0.0276 | 0.0167 | 0.081 | 0.0109 | -0.0642 | -0.1426 | -0.2506 | -0.2088 |
|  | Not being able to do things that other children his or her age can do | **-0.3524*** | -0.0717 | -0.0593 | **-0.3441*** | -0.1227 | -0.1555 | **-0.3127*** | **-0.4190*** | **-0.3034*** |
|  | Keeping up when playing with other children | -0.1819 | -0.0124 | -0.176 | -0.1807 | -0.0122 | -0.2079 | **-0.3168*** | **-0.3346*** | **-0.3068*** |
| School | Doing the same daycare preschool kindergarten school activities as other children his or her age | -0.2877 | -0.0654 | -0.1092 | -0.0826 | 0.1068 | -0.2767 | -0.2578 | **-0.3905*** | **-0.4685*** |
|  | Missing school because of not feeling well | 0.1206 | -0.1723 | **-0.3318*** | -0.1975 | -0.166 | -0.1968 | -0.283 | -0.2839 | **-0.3705*** |
|  | Missing school to go to the doctor or hospital | 0.0227 | -0.2447 | **-0.3136*** | -0.27 | -0.2617 | -0.0701 | -0.2638 | -0.1321 | -0.2712 |
| Correlation coefficients were calculated by Spearman rank correlation. *: correlation significant at 0.05 level..  High correlations, ≥ 0.5 (green); moderate correlations, 0.3–0.49 (orange); low correlation, < 0.3 (black).  Grey shaded cell indicates expected moderate or high correlations (r ≥ 0.3) based on highly similar items in line with published technical guide  PF: physical functioning ; PS: physical symptoms; EF: emotional functioning; SF: social-functioning. School functioning | | | | | | | | | | |

| Table S11 Convergence between CHU9D dimensions and PedsQL items in children aged 5-7 years | | | | | | | | | | |
| --- | --- | --- | --- | --- | --- | --- | --- | --- | --- | --- |
| PedsQL (5-7 years) | | **CHU9D** | | | | | | | | |
|  |  | Worried | Sad | Pain | Tired | Annoyed | School | Sleep | Daily routine | Activities |
| PF | Walking more than one block | -0.109 | -0.1906 | -0.2799 | -0.2433 | -0.2494 | **-0.4196*** | **-0.3294** | **-0.4234*** | **-0.4645*** |
|  | Running | -0.0936 | -0.2242 | -0.2703 | -0.2275 | -0.2571 | **-0.4207*** | **-0.325** | **-0.4332*** | **-0.4697*** |
|  | Participating in sports activity or exercise | -0.2159 | -0.2447 | **-0.3514** | -0.1978 | **-0.3423** | **-0.5963*** | **-0.4285*** | **-0.5028*** | **-0.5223*** |
|  | Lifting something heavy | 0.0236 | **-0.3451** | -0.1291 | -0.0207 | -0.2616 | **-0.3349** | -0.0081 | -0.2119 | -0.2095 |
|  | Taking a bath or shower by him or herself | **-0.3705*** | -0.2719 | -0.0593 | -0.1439 | -0.2084 | **-0.4695*** | **-0.3181** | **-0.5741*** | **-0.3635*** |
|  | Doing chores, like picking up his or her toys | -0.0858 | -0.2261 | -0.2144 | -0.1021 | **-0.4760*** | **-0.3655*** | **-0.4120*** | **-0.4173*** | -0.2538 |
|  | Having aches or pains | -0.1026 | -0.2032 | -0.2859 | -0.0107 | **-0.331** | **-0.3606*** | -0.2865 | **-0.5251*** | **-0.4182*** |
|  | Having a low energy level | -0.1224 | **-0.3399** | -0.111 | **-0.4209*** | -0.2757 | **-0.4041*** | -0.2411 | **-0.5835*** | **-0.4076*** |
| EF | Feeling afraid or scared | -0.1604 | -0.2674 | 0.0618 | -0.1488 | -0.222 | -0.2831 | **-0.3364** | **-0.3247** | -0.059 |
|  | Feeling sad | -0.0904 | -0.1803 | -0.1017 | **-0.3255** | -0.2241 | -0.2268 | -0.2515 | -0.1214 | 0.0599 |
|  | Feeling angry | -0.2252 | -0.1343 | -0.181 | -0.01 | **-0.5583*** | -0.1985 | **-0.4502*** | **-0.3133** | **-0.3879*** |
|  | Having trouble sleeping | -0.2239 | -0.2671 | **-0.3074** | -0.1686 | **-0.3928*** | **-0.4084*** | **-0.8498*** | **-0.5043*** | **-0.4773*** |
|  | Worrying about what will happen to him or her | -0.2404 | 0.0514 | 0.1169 | -0.2617 | -0.1777 | -0.2456 | **-0.3476** | -0.1991 | **-0.3570*** |
| SF | Getting along with other children | -0.1221 | **-0.3416** | -0.0283 | -0.1321 | **-0.6059*** | **-0.4430*** | **-0.3245** | **-0.4740*** | **-0.5219*** |
|  | Other children not wanting to to be his or her friend | 0.0347 | 0.0246 | -0.0143 | -0.0761 | **-0.302** | -0.2845 | **-0.5615*** | **-0.3187** | **-0.4388*** |
|  | Getting teased by other children | 0.0204 | -0.0468 | 0.2361 | -0.028 | **-0.4325*** | -0.2839 | -0.2994 | **-0.3859*** | **-0.3109** |
|  | Not being able to do things that other children his or her age can do | -0.1457 | **-0.3984*** | -0.2334 | **-0.3099** | **-0.5965*** | **-0.5138*** | **-0.5021*** | **-0.5546*** | **-0.3528*** |
|  | Keeping up when playing with other children | -0.1347 | -0.2131 | -0.1875 | -0.2377 | **-0.3724*** | **-0.4358*** | **-0.4270*** | **-0.7365*** | **-0.6530*** |
| School | Paying attention in class | -0.0863 | -0.1959 | -0.1317 | -0.2946 | -0.2906 | **-0.4095*** | **-0.6057*** | **-0.4154*** | **-0.4204*** |
|  | Forgetting things | -0.0761 | -0.0888 | -0.1477 | -0.2892 | **-0.3393** | **-0.3466*** | **-0.4017*** | **-0.3544*** | **-0.3486*** |
|  | Keeping up with school activities | -0.1801 | **-0.3758*** | -0.2848 | **-0.4618*** | **-0.4157*** | **-0.5512*** | **-0.6968*** | **-0.6706*** | **-0.4843*** |
|  | Missing school because of not feeling well | -0.1308 | **-0.3234** | -0.0761 | -0.2797 | **-0.3492*** | **-0.4847*** | **-0.3229** | **-0.5022*** | **-0.4323*** |
|  | Missing school to go to the doctor or hospital | -0.0982 | -0.117 | -0.1329 | -0.0245 | **-0.3227** | **-0.3802*** | -0.1582 | **-0.4186*** | -0.2179 |
| Correlation coefficients were calculated by Spearman rank correlation. *: correlation significant at 0.05 level. High correlations, ≥ 0.5 (green); moderate correlations, 0.3–0.49 (orange); low correlation, < 0.3 (black). Grey shaded cell indicates expected moderate or high correlations (r ≥ 0.3) based on highly similar items in line with published technical guide  physical functioning ; PS: physical symptoms; EF: emotional functioning; SF: social-functioning. School functioning | | | | | | | | | | |

| Table S12 CHU9D utilities and PedsQL total scores for children with colorectal conditions age 2 years and above | | | | | | |
| --- | --- | --- | --- | --- | --- | --- |
|  | CHU9D (Australian adolescent value set) | | CHU9D (UK value set) | | PedsQL | |
|  | mean | SD | mean | SD | mean | SD |
| 2-4 years |  |  |  |  |  |  |
| ARM or HD | 0.74 | 0.19 | 0.87 | 0.09 | 81.30 | 14.87 |
| ARM | 0.79 | 0.16 | 0.89 | 0.07 | 82.88 | 13.24 |
| HD | 0.66 | 0.23 | 0.84 | 0.10 | 78.40 | 17.51 |
| 5-7 years |  |  |  |  |  |  |
| ARM or HD | 0.68 | 0.23 | 0.83 | 0.12 | 74.17 | 18.91 |
| ARM | 0.67 | 0.19 | 0.84 | 0.11 | 77.67 | 17.00 |
| HD | 0.68 | 0.26 | 0.82 | 0.14 | 71.22 | 20.36 |
| ARM: anorectal malformations; HD: Hirschsprung disease. SD: standard deviation | | | | | | |


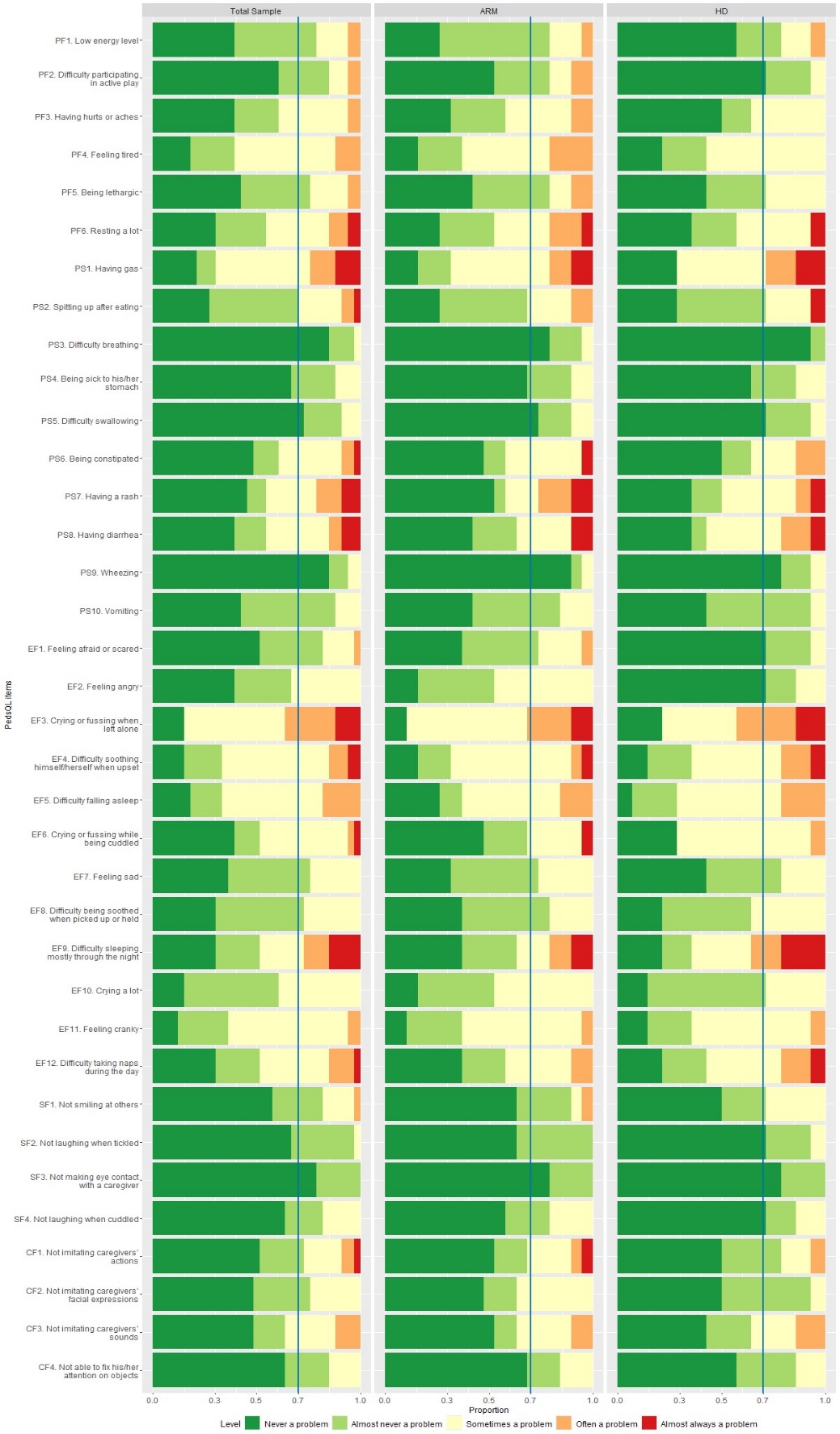


Figure S1. Response distribution on PedsQL items in children age 1-12 months and by condition.

Legend: ARM: anorectal malformations; HD: Hirschsprung disease.


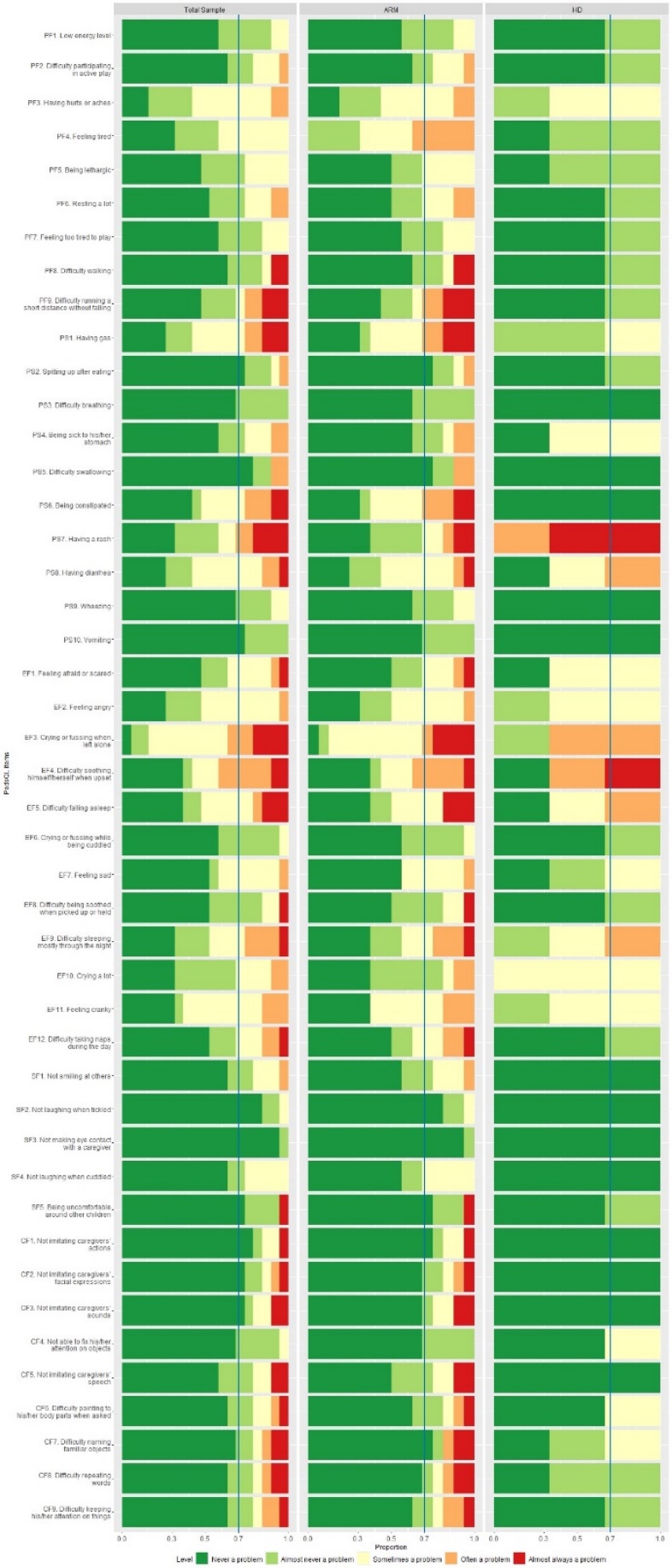


Figure S2. Response distribution on PedsQL items in children age 13-24 months and by condition.

Legend: ARM: anorectal malformations; HD: Hirschsprung disease.


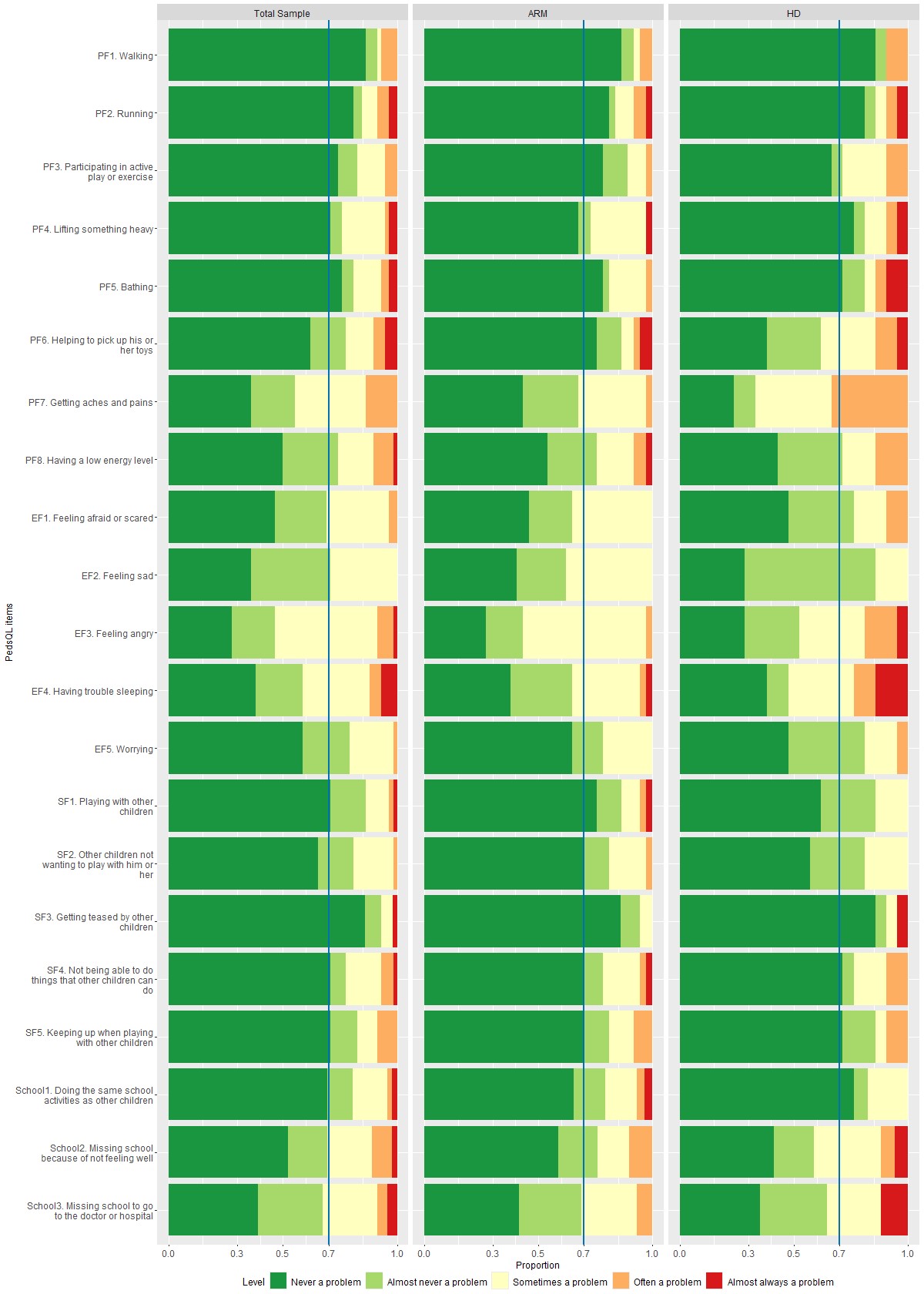


Figure S3. Response distribution on PedsQL items in children age 2-4 years and by condition.

Legend: ARM: anorectal malformations; HD: Hirschsprung disease.


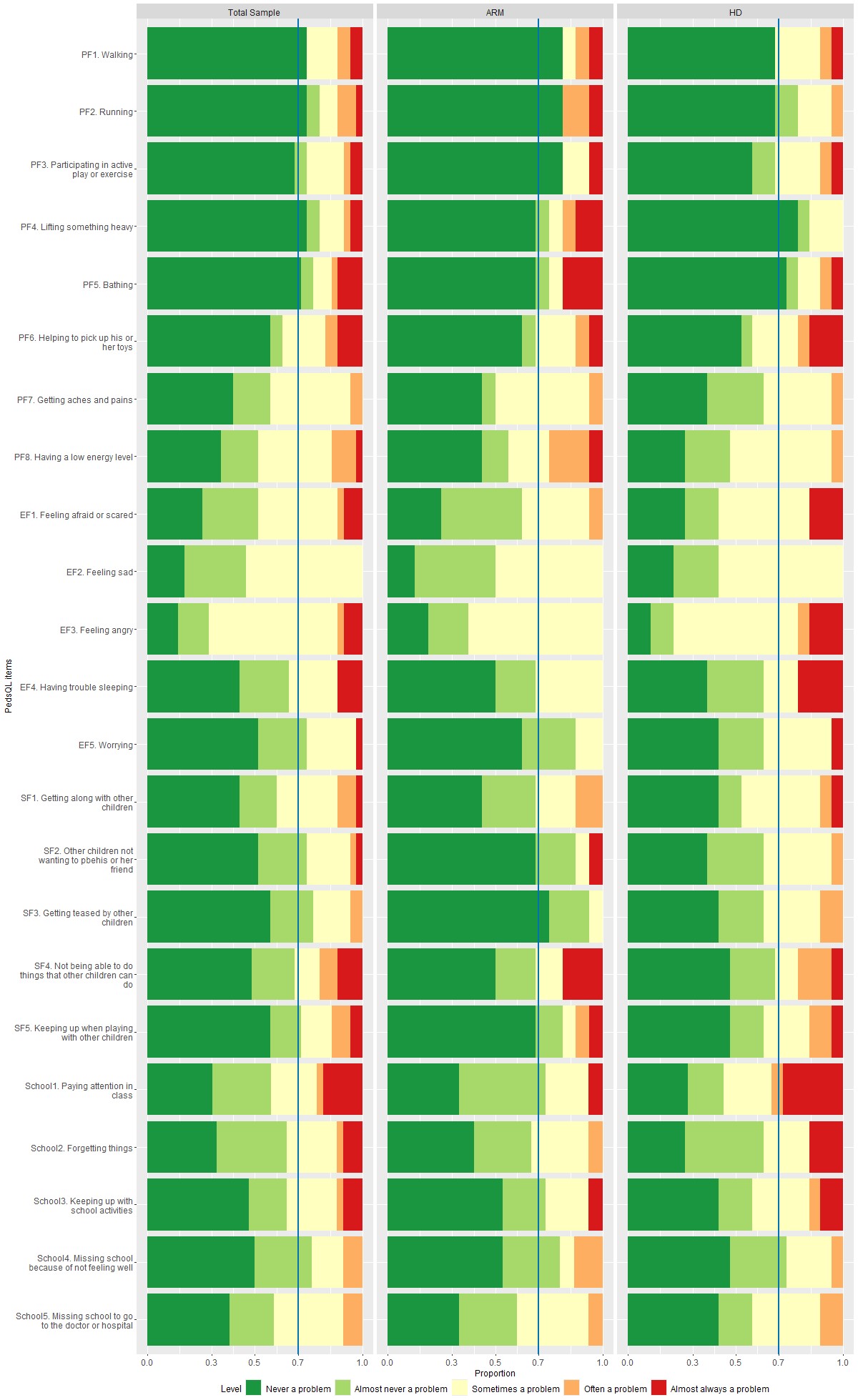


Figure S4. Response distribution on PedsQL items in children age 5-7 years and by condition.

Legend: ARM: anorectal malformations; HD: Hirschsprung disease.
